# Graphene Oxide Catalyzed Synthesis of Fused Chromeno Spiro Pyrrolidine Oxindoles *via* Tandem Decarboxylation and 1,3-Dipolar Cycloaddition

**DOI:** 10.3389/fchem.2021.759436

**Published:** 2022-01-11

**Authors:** Vipin Singh, Shanta Raj Lakshmi, L. Raju Chowhan

**Affiliations:** Centre for Applied Chemistry, Central University of Gujarat, Gandhinagar, India

**Keywords:** chromeno spirooxindoles, azomethine ylides, heterogeneous catalysis, multi component reactions, 1,3 dipolar cycloaddition, coumarin

## Abstract

A short and efficient multicomponent sequence for synthesizing fused novel polyheterocyclic chromeno spiro-pyrrolidine oxindoles *via* 1,3-dipolar cycloaddition reaction mediated by reactive azomethine ylides catalyzed by the Graphene Oxide (GO) is reported herein. This approach was utilized for synthesizing fused polyheterocyclic spiro-pyrrolothiazole and spiro-pyrrole oxindoles with yields ranging from good to excellent. A heterogeneous GO catalyst with an ultra-low catalytic loading of 0.05 wt% could proficiently catalyze the reaction without the formation of any side products and can also be visualized by the formation of solid mass in the reaction flask. The methodology is green in nature and the products were isolated by simple filtration without the use of any chromatographic techniques.

## Introduction

Spiro-heterocycles form a major class of natural products and have good biological activities. The chiral spiro carbon leads to sterically constrained spiro structures, which probably explains the wide scope of pharmacological activities. The spirooxindoles nucleus is found in numerous natural products and often dictates biological activities like anti-cancer, anti-viral, anti-inflammatory, anti-leukemic, and anti-tubercular effects, to name a few.

Cycloaddition reactions are one of the most frequented classes of reactions in organic chemistry. In synthetic chemistry, isatin has a highly reactive C-3 carbonyl group and complex multi-spiro-heterocycles can be constructed by implying 1,3-dipolar cycloaddition reactions (Borad et al., 2014; Zhu et al., 2017; Dwivedi et al., 2021), Morita–Baylis–Hillman reaction (Deng et al., 2011; Liao et al., 2021), electrocyclization (Viswambharan et al., 2010), and photo-induced reactions (Wang et al., 2005). The 1,3-dipolar cycloaddition reactions (1,3-DCR) are a fascinating set of protocols that are often regarded as the most competent process and opens new vistas in the field of furnishing efficient and high-yield products in a regio- and stereo-controlled fashion (Coldham and Hufton, 2005; Ramesh et al., 2018; Reddy et al., 2018a; Reddy et al., 2018b; Kumar et al., 2019; ).

An important aspect that further establishes the importance of 1,3-DCR is the placement of the ylide dipole and alkene or alkyne dipolarophile within the same molecule, which can provide direct access to polycyclic compounds with extensive complexity and interesting architecture (Padwa and Pearson, 2003; Borah et al., 2021a; Borah et al., 2021b; Borah et al., 2021b). Various methods for cycloaddition reactions involve the formation of highly reactive azomethine ylides and the addition of suitable dipolarophiles; the success of this setup is often described in the synthesis of pyrrolidine and pyrrole containing natural products and medicinally important synthetic targets (Namboothiri and Hassner, 2001) Figure 1. The conformational constraints and juxtaposition of reactants frequently lead to readily cycloaddition with complete or very high selectivity. For the reasons mentioned above, 1,3-DCR has been described as “the single most important method for the construction of heterocyclic five-membered rings” (Jørgensen, 2002; Gupta and Khurana., 2019; Thadem et al., 2021).

**FIGURE 1:**
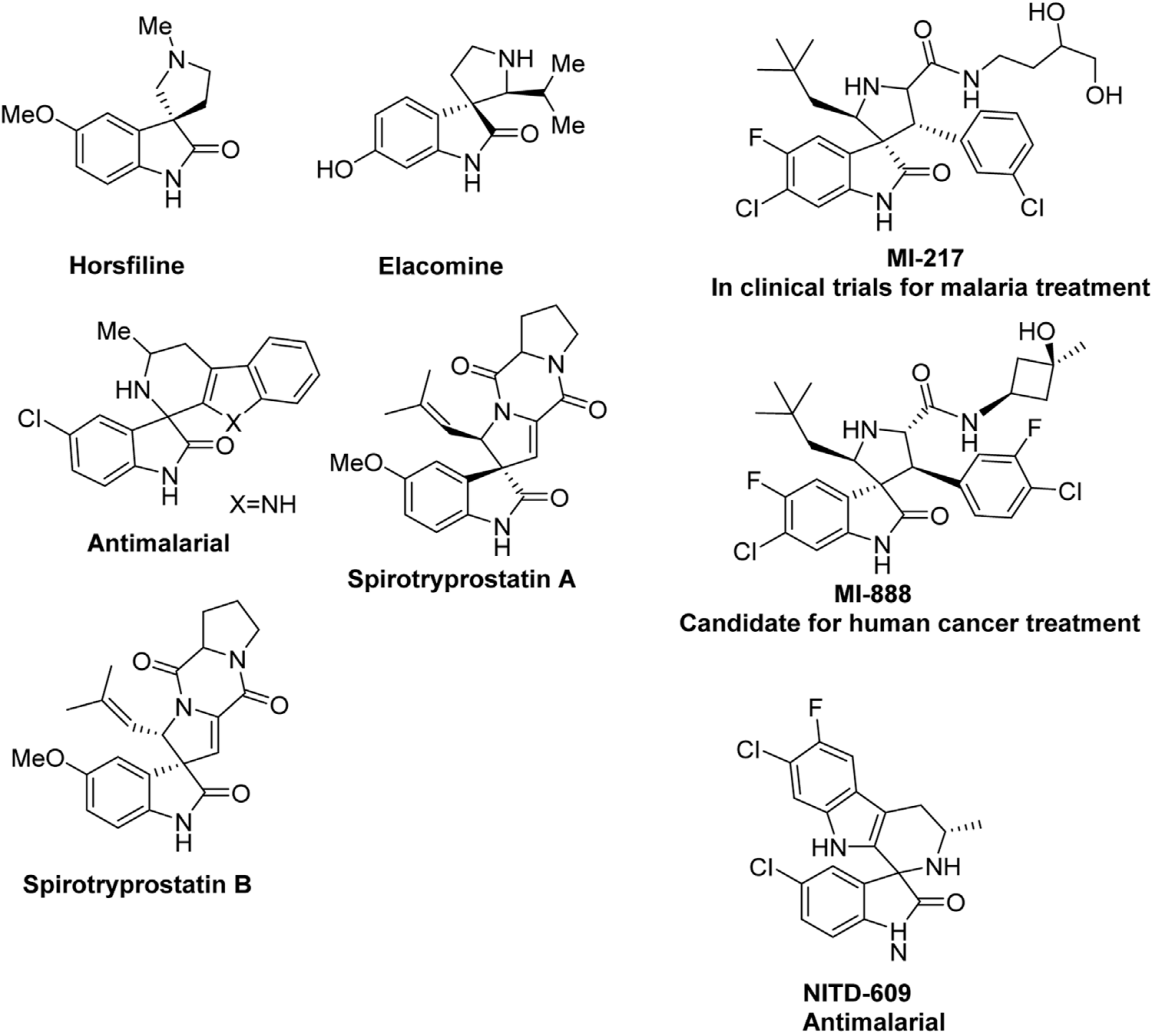
Some important naturally occurring and synthetic spirooxindoles containing molecules.

Major hurdles that regularly occur in organic synthesis are atom economy and novel and efficient reactions, which can answer both the ecological aspect and target-oriented synthesis (de Graaff et al., 2012; Lledó et al., 2019). Often traditional synthesis routes include multiple steps and long, tedious purification processes like column chromatography; these stages often add a significant amount of side products and also generate a huge amount of waste. One probable answer to the ever-growing challenge of environment pollution is multicomponent reactions (MCRs) that are widely regarded as a dynamic tool in organic chemistry as they have a wide scope of tolerance for various functional groups. MCRs often fascinate chemists by their ability to create a library of molecules with considerable complexity and geometry, which is otherwise rarely encountered in synthesis. Another aspect that points to the environmental conscience is the usage of commercially available reactants in one-pot conversion to a complex/target molecule (Xu et al., 2016; Kumar et al., 2021). This conversion often includes the formation of multiple bonds in a single step, thereby including high atom economy, operational simplicity, and considerably less requirement of energy, which further proves MCRs as the perfect candidate for the synthesis of natural products, structurally diverse, and pharmaceutically active compounds (Sunderhaus and Martin, 2009).

## Results and Discussion

MCRs being operationally simple, if coupled with heterogeneous catalysts like graphene oxide (GO), can result in sequences that are easy to carry out with high yields (Sachdeva., 2020; Naeim-Fallahiyeh et al., 2020). Currently, the synthesis of complex molecules having appealing stereochemistry being synthesized by relatively simple protocol and environmental conscience is of utmost importance and can be largely correlated by frequent reports occurring in literature (Sharma et al., 2021; Singh et al., 2021). As a part of our group’s research interest in sustainable and operationally simple, efficient sequences, we wish to report a simple and effective method mediated by azomethine ylide for the construction of a library of complex spiro-pyrrolizineindoline and spiro-pyrroleindoline molecules *via* 1,3-dipolar cycloaddition reaction in a one-pot, multicomponent approach using a heterogeneous catalyst (Chowhan et al., 2019; Lakshmi et al., 2020).

**SCHEME 1:**
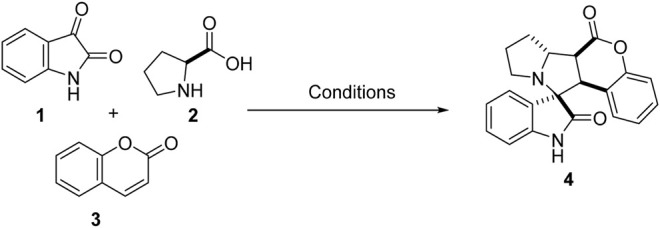
Optimization of reaction conditions.

**SCHEME 2:**
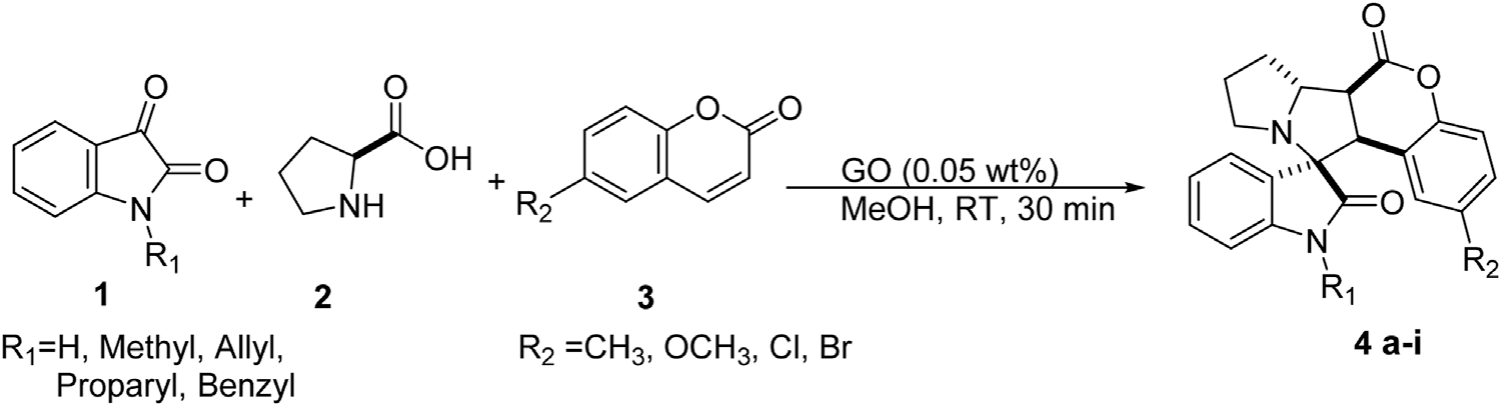
Reaction conditions for the synthesis of spiro pyrrolizineindoline.

Our synthetic approach started with 1,3-dipolar cycloaddition of α,β-unsaturated carbonyl compounds in equimolar concentrations, i.e., coumarin **3** and non-stabilized azomethine ylide, *in situ* generated *via* the condensation of isatin **1** and L-proline **2** in protic solvents (Scheme 1; Table 1). The mixture was stirred vigorously at room temperature and the progress of the reaction was monitored continuously by thin-layer chromatography (TLC). After 24 h, we observed that the yields of 4 (18%) were appreciable but not satisfactory (Entry 1, Table 1). MeOH used as solvent gave somewhat better yields of 22% (Entry 2, Table 1). Owing to the large surface area and acidic nature of GO, GO 0.5% by wt. was then used as a catalyst and to our delight, the reaction proceeded swiftly to afford a single product **4** in 97% yields (Entry 3, Table 1). To assess even better yields, catalytic loading was then increased to 1 and 2% by wt.; however, significant improvements in yields, 94% and 95%, respectively, were not observed (Entry 4–5, Table 1 **)**. Intrigued by the results, we then applied this protocol to a series of isatins **1**, L-proline **2,** and coumarin **3** to create a series of spirocyclo adducts (Scheme 2; Table 2). It is worth mentioning that further reduction in catalytic loading led to a slight decrease in the yields with increased reaction times (Entry 3, Table 1). Another aspect is that all the products were obtained in analytically pure form and did not require filtration by column chromatography and the formation of the product can be visualized by the formation of solid mass in the reaction flask. Solid products were collected on filter paper, dissolved in ethyl acetate, and passed through a pad of celite to filter out the heterogeneous catalyst followed by removing the solvent under reduced pressure and washing it with cold methanol.

**TABLE 1:** Optimization of reaction conditions.

Entry^a^	Solvent	Graphene Oxide (GO) wt%	Time	Yield %
1	EtOH	—	24 h	18%
2	MeOH	—	24 h	22%
3	MeOH	0.5	10 min	97%
4	MeOH	1	10 min	94%
5	MeOH	2	10 min	95%
6	MeOH	0.25	1 h	87%

a All reactions were carried out at 0.5 mmol scale, with 1 eq. of isatin, 1 eq. of proline, 0.5 wt% GO, and 1 eq. coumarin at rt.

**TABLE 2:** Substrate scope for spiro pyrrolizineindoline synthesis.

	
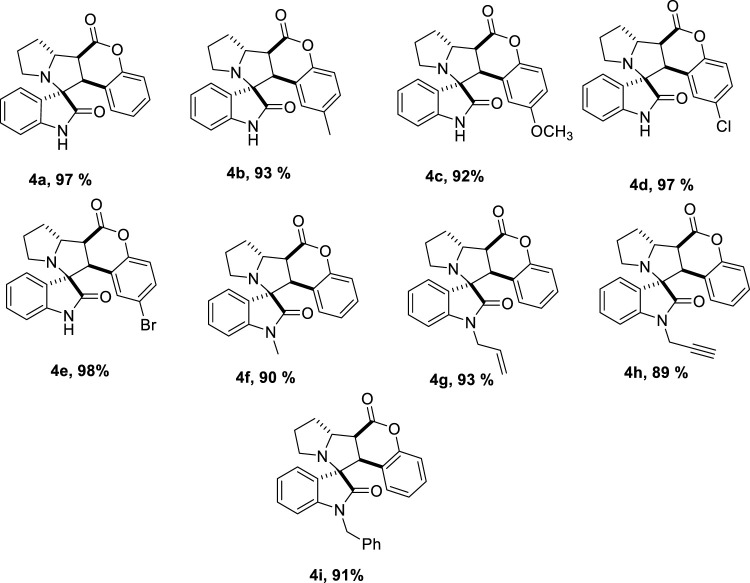	

Optimized reaction conditions could efficiently utilize a wide range of substrates (Scheme 2). Coumarins, when substituted with an electron donating group, the yield decreased slightly (Table 2, 4a *vs.* 4b, 4c), 95% to 93 and 92%, respectively. Upon the substitution of an electron withdrawing group on coumarin, the yield increased from 95% in (**4a**) to 97% in (**4d**) and 98% (**4e**). N- substituted isatins generally gave good to excellent yields.

**SCHEME 3:**
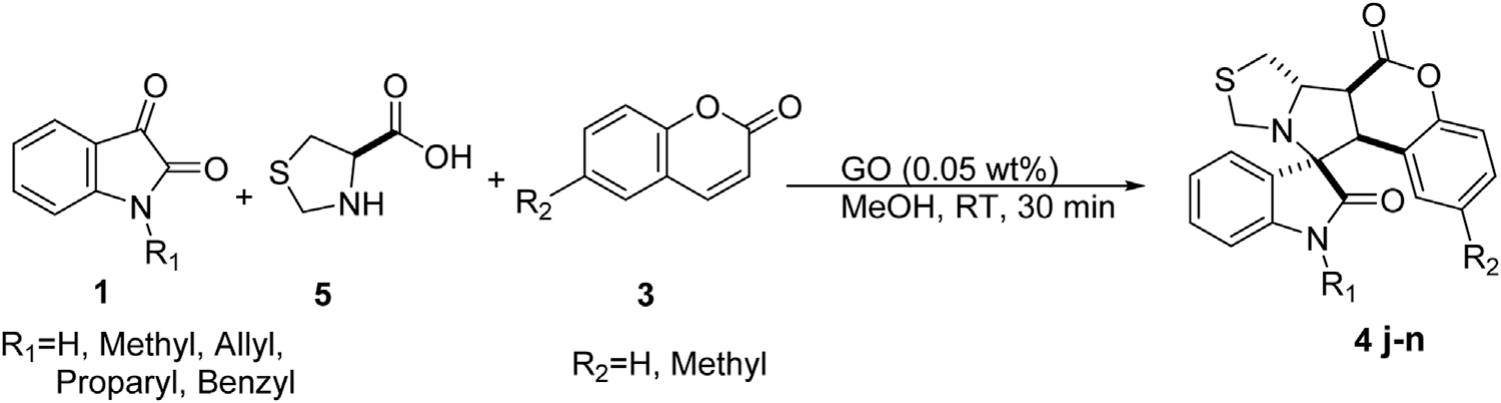
Reaction conditions for the synthesis of spiro pyrrolothiazoleindoline.

To further establish the scope of the methodology, proline was replaced with thio-proline **5**; again, the expected product **4j-n** was obtained in good to excellent yields, as shown in Scheme 3; Table 3.

**TABLE 3:** Substrate scope for spiro pyrrolothiazoleindoline synthesis.

	
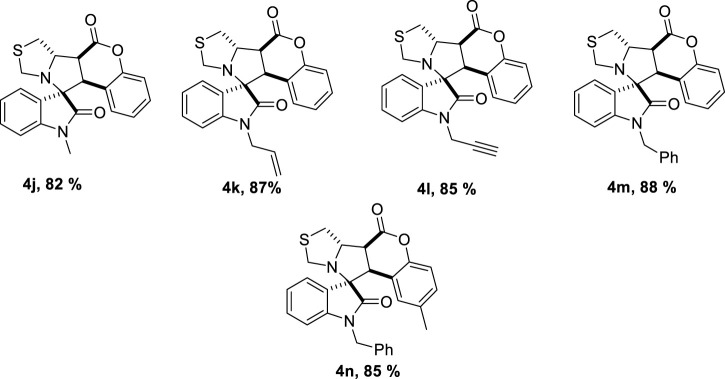	

**SCHEME 4:**
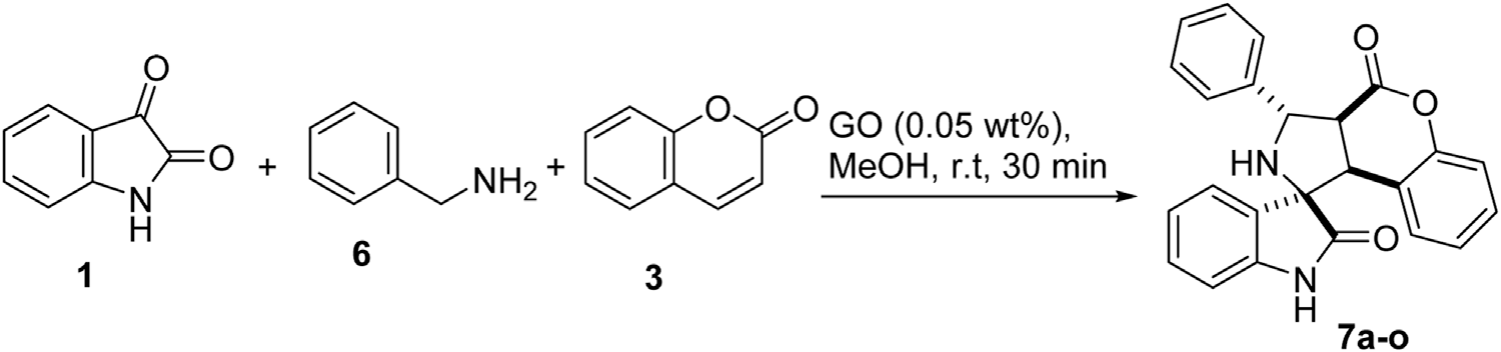
Reaction conditions for the synthesis of spiro pyrroleindoline.

With these optimized conditions, we were intrigued to explore the substrate scope of the methodology further; to our delight, we found that the reaction conditions could further accommodate a wide range of various substituted benzyl amines 6 (Scheme 4). It is worth mentioning that benzylamines are less reactive and according to a previous report, benzylamine fails to give the desired product (Liu et al., 2016). However, under optimized conditions, a series of compounds were synthesized with yields ranging from good to excellent. In this case, also the products could be obtained analytically pure without involving column chromatography (Table 4).

**TABLE 4:** Substrate scope for the synthesis of spiro pyrroleindoline.

	
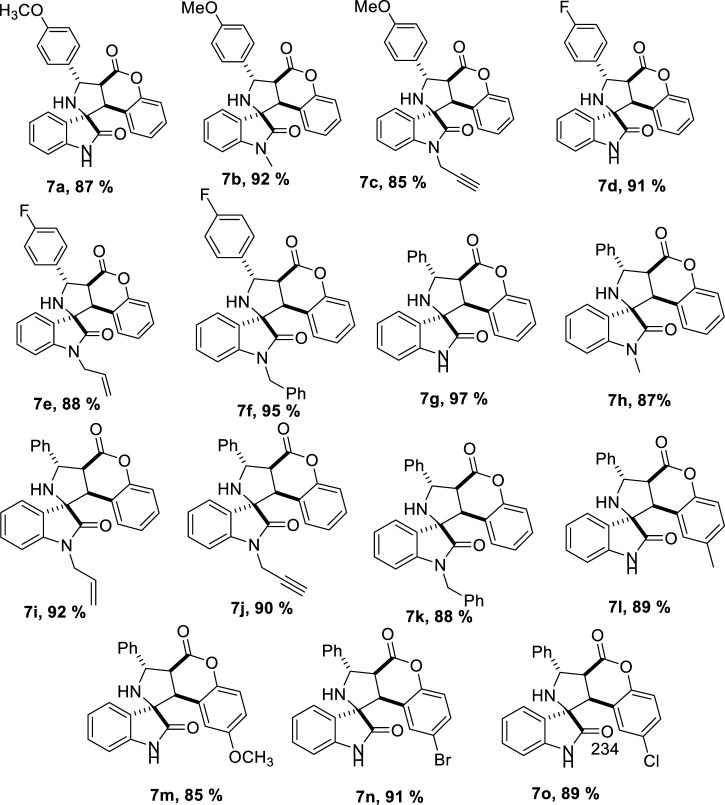	

To strengthen the efficacy of the established reaction conditions, a series of reactions were planned using the optimized reaction conditions with various substituted isatins, benzyl amines, and coumarins. Methyl substituted isatin **7b** gave a slightly better 92% yield, as compared to unsubstituted (**7b**, 87%), allyl (**7e**, 88%), and propargyl (**7c**, 85%) substituted isatins. Benzyl amine substituted with the electron withdrawing group (fluorine) **7d**, 91%, gave somewhat better results than amines substituted with the electron donating group (methoxy) **7a**, 87%. Methoxy substituted coumarin underperformed with 85% yield, **7m**, and bromo substituted coumarin gave better results (**7n**, 91%). It can be inferred that with the substitution of the electron donating groups, the yield generally decreased, whereas with the substitution of the electron withdrawing groups, the reaction performed slightly better.

### Plausible Mechanism

**FIGURE 2:**
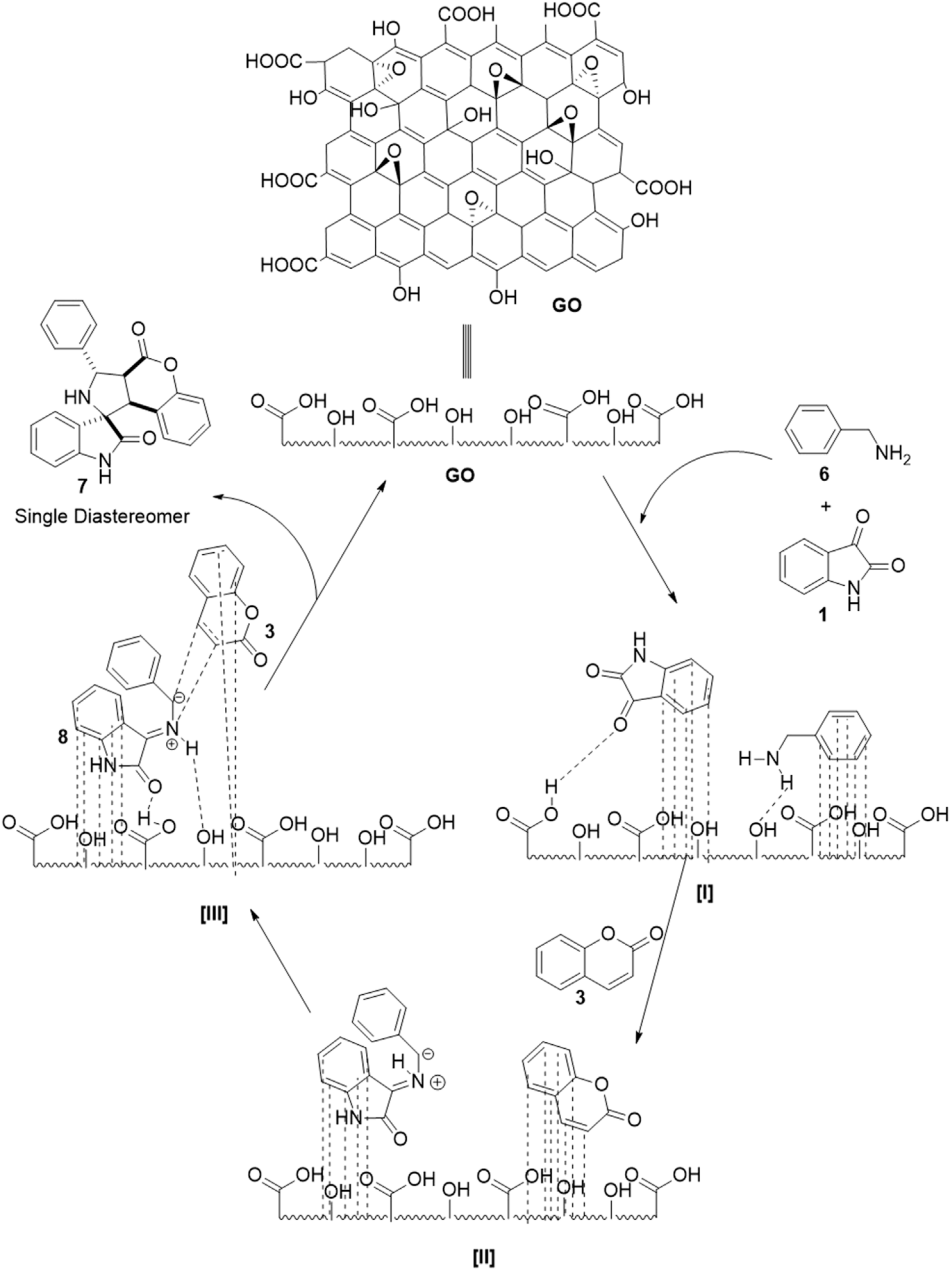
Plausible mechanism for GO catalyzed synthesis of spiro pyrroleindoline.

We assume the following plausible mechanism for the reaction (Figure 2). Reactants are initially localized onto the surface of the catalyst owing to π-stacking and the capability of the catalyst to form hydrogen bonding **I** and **II** (Reddy et al., 2018a; Reddy et al., 2018b). Due to the acidic nature of GO, it enables the imine formation to generate very reactive azomethine ylide **8** (intermediate **II**). Due to the sheet form of GO, the dipolarophile can approach the reactive azomethine ylide from one side exclusively, which yields a single diastereomer as the product **III**. The interaction between heteroatoms in the oxindole motif with the azomethine ylide dipole (zwitterion) generated during reaction directs the orientation of incoming dipolarophile (coumarin) onto the dipole **III**.

## Conclusion

In conclusion, we have explored one-pot multicomponent 1,3-dipolar cycloaddition reaction mediated by azomethine ylide catalyzed by heterogeneous catalyst sequence for the diastereoselective and regioselective synthesis of spirochromeno pyrrolizine/pyrrolothiazole/pyrrole -indolinedione. Single-step, facile synthesis and ultra-low catalytic loading with direct access to pure products in good to excellent yields simply by filtration establishes the importance and hence usability of this practical approach. The protocol as an extension of frequently used MCRs with simple sequence and sort reaction times deserves some consideration and attention in synthetic chemistry.

## Data Availability

The original contributions presented in the study are included in the article/Supplementary Material; further inquiries can be directed to the corresponding author.
