# Supplementary material for: Graphene Oxide Catalyzed Synthesis of Fused Chromeno Spiro Pyrrolidine Oxindoles via Tandem Decarboxylation and 1,3-Dipolar Cycloaddition
Source: Front Chem. 2022 Jan 11;9:759436. doi: 10.3389/fchem.2021.759436 (PMC8787336; doi:10.3389/fchem.2021.759436)
Supplement: Supplementary file 1 [file DataSheet1.docx]

Supplementary Material

**Graphene Oxide Catalysed Synthesis of Fused Chromeno Spiro Pyrrolidine Oxindoles via Tandem Decarboxylation and 1,3 Dipolar Cycloaddition**

Vipin Singh,*^a^* Shanta Raj Lakshmi,*^a^* L. Raju Chowhan*^,^*^a^*

^a^ Vipin Singh, Dr. Shanta Raj Lakshmi, Dr. L. Raju Chowhan*, Centre for Applied Chemistry, Central University of Gujarat, Sector 30, Gandhinagar-382030, India.

Corresponding Author:

E-mail: rchowhan@cug.ac.in

**Table of Contents**

**1.** General Experimental Details……………………………………………............................2

**2.** Spectral Data of Products..................................................................................................3 -16

**3**. Copies of ^1^H NMR and ^13^C NMR Spectra.....................................................,,..............17-47

**1.** **General Experimental Details**

**1.1 General Information**

Flasks were oven or flame-dried and cooled in a desiccator. All reactions were carried out under an ambient atmosphere. Thin-layer chromatography was performed on Merck TLC Silica gel 60 F_254_ silica gel plates. The spots were visualized with ultraviolet light and/or *p*-Anisaldehyde with heat as revealing agent. ^1^H NMR and ^13^C NMR were recorded at 500 and 126 MHz, respectively, and chemical shifts are based on TMS peak at *δ* = 0.00 pm for proton NMR and CDCl_3_ peak at *δ* = 77.00 ppm (t) in carbon NMR. IR characterization data were recorded on a FT-IR spectrometer (Perkin Elmer) FT/IR-5700. High-resolution mass spectra were obtained using positive electrospray ionization by TOF method. Multiplicity is indicated as follows: s (singlet); bs (broad singlet); d (doublet); t (triplet); q (quartet); m (multiplet); dd (doublet of doublets), etc. Reagents were purchased from commercially suppliers (Alfa Aesar, Sigma-Aldrich or Avra Synthesis) and used as received, unless stated otherwise.

- 1. **General procedure**

A mixture of isatins **1** (73.5 mg, 0.5 mmol) and prolines **2** (57.6 mg, 0.5 mmol**) /5** (66.6mg, 0.5 mmol) or benzyl amine **6** (53.6 mg, 0.5 mmol) and coumarins **3** (73 mg, 0.5 mmol) was added to stirring suspension of graphene oxide (3.7 mg, 0.50 wt.% in MeOH) in methanol as solvent and stirred under ambient air atmosphere for 10-30 min. Reaction can be visualized by the change of color change in the reaction pot. The progress of the reaction was monitored by TLC. After completion of reaction the reaction mixture was filtered through filter paper and the solid product collected was dissolved in EtOAc 20 mL then passed through pad of celite. The organic layer was washed with brine, dried over anhydrous Na_2_SO_4_. The solvent was evaporated under vacuum to afford crude solid which was further purified by washing with 2 x 5 mL cold methanol to afford pure products **4a-n/7a-o** as colourless to pale yellow solids.

**2.** **Spectral Data of Products**

**6a,6b,7,8,9,11a-hexahydro-6H-spiro[chromeno[3,4-a]pyrrolizine-11,3'-indoline]-2',6-dione (4a):** 167.9 mg, pale yellow solid. TLC: R*_f_* = 0.67 (60% EtOAc/Hexanes); M. P. 196-198 °C. IR (KBr) 3213, 3033, 2964, 2873, 2426, 1471, 1749, 1714, 1638, 1619, 1471, 1361, 1099, 976; ^1^H NMR (500 MHz, DMSO-*d*_6_) *δ* 10.09 (s, 1H), 7.76 (d, *J* = 7.4 Hz, 1H), 7.31 (t, *J* = 7.6 Hz, 1H), 7.20 - 7.10 (m, 2H), 6.97 (d, *J* = 8.1 Hz, 1H), 6.78 (t, *J* = 7.4 Hz, 1H), 6.71 (d, *J* = 7.6 Hz, 1H), 6.31 (d, *J* = 7.6 Hz, 1H), 4.38 (s, 1H), 4.15 (d, *J* = 11.3 Hz, 1H), 3.15 (d, *J* = 12.2 Hz, 1H), 2.61 (t, *J* = 6.9 Hz, 1H), 2.36 (dd, *J* = 18.9, 8.2 Hz, 1H), 1.92 - 1.78 (m, 2H), 1.71 – 1.59 (m, 1H); ^13^C NMR (126 MHz, DMSO-*d*_6_) *δ* 177.49, 167.65, 150.86, 143.37, 129.95, 128.59, 127.48, 126.72, 126.15, 123.44, 121.66, 118.00, 116.51, 109.78, 76.45, 67.07, 50.41, 47.05, 42.31, 32.82, 26.04; HRMS (ESI+): *m/z* calculated for [C_21_H_18_N_2_O_3_ + H]^+^ : 347.13956; found: 347.14289.

**6a,6b,7,8,9,11a-hexahydro-6H-spiro[chromeno[3,4-a]pyrrolizine-11,3'-indoline]-2',6-dione (4b):** 167 mg, pale yellow solid. TLC: R*_f_* = 0.71 (60% EtOAc/Hexanes); M. P. 200-203 °C. IR (KBr) 3206, 3033, 2426, 1748, 1715, 1637, 1619, 1471, 1416, 1348, 1325, 974; ^1^H NMR (500 MHz, DMSO-*d*_6_) *δ* 10.07 (s, 1H), 7.75 (d, *J* = 7.2 Hz, 1H), 7.32 (t, *J* = 7.4 Hz, 1H), 7.13 (t, *J* = 7.2 Hz, 1H), 6.96 (d, *J* = 7.8 Hz, 1H), 6.85 (d, *J* = 8.0 Hz, 1H), 6.72 (d, *J* = 7.4 Hz, 1H), 6.05 (s, 1H), 4.38 (s, 1H), 4.11 (dd, *J* = 23.7, 8.0 Hz, 2H), 3.32 (s, 1H), 3.17 (d, *J* = 4.0 Hz, 3H), 2.62 (s, 1H), 2.35 (d, *J* = 9.9 Hz, 1H), 1.82 (m, 2H), 1.66 (s, 1H); ^13^C NMR (126 MHz, DMSO-*d*_6_) *δ* 178.64, 168.31, 148.89, 142.89, 133.35, 130.27, 129.75, 127.92, 125.86, 125.84, 122.30, 116.84, 116.62, 111.11, 67.77, 51.26, 50.62, 47.93, 43.85, 33.99, 26.63, 20.62; HRMS (ESI+): *m/z* calculated for [C_22_H_20_N_2_O_3_ + H]^+^ : 361.15521; found: 361.15722.

**2-methoxy-6a,6b,7,8,9,11a-hexahydro-6H-spiro[chromeno[3,4-a]pyrrolizine-11,3'-indoline]-2',6-dione (4c):** 173 mg, pale yellow solid. TLC: R*_f_* = 0.48 (60% EtOAc/Hexanes); M. P. 205-207 °C. IR (KBr) 3328, 3039, 2843, 1725, 1636, 1619, 1484, 1363, 1347, 1299, 1253, 956; ^1^H NMR (500 MHz, DMSO-*d*_6_) *δ* 8.01 (s, 1H), 7.54 (d, *J* = 7.4 Hz, 1H), 7.34 (t, *J* = 7.7 Hz, 1H), 7.16 (t, *J* = 7.4 Hz, 1H), 6.88 (d, *J* = 8.8 Hz, 1H), 6.83 (d, *J* = 7.8 Hz, 1H), 6.67 (d, *J* = 8.9 Hz, 1H), 5.77 (s, 1H), 4.74 (s, 1H), 3.88 (d, *J* = 11.2 Hz, 1H), 3.36 (s, 3H), 3.09 (d, *J* = 11.0 Hz, 2H), 2.94 (t, *J* = 6.9 Hz, 1H), 2.67 – 2.59 (m, 1H), 1.98 (d, *J* = 5.9 Hz, 1H), 1.93 – 1.75 (m, 2H); ^13^C NMR (126 MHz, DMSO-*d*_6_) *δ* 176.34, 166.55, 153.69, 143.50, 142.44, 128.58, 124.91, 120.48, 116.08, 113.81, 109.53, 108.98, 75.51, 66.42, 53.63, 49.63, 46.09, 42.41, 32.20, 25.11; HRMS (ESI+): *m/z* calculated for [C_22_H_20_N_2_O_4_ + H]^+^ : 377.15013; found: 377.15364.

**2-chloro-6a,6b,7,8,9,11a-hexahydro-6H-spiro[chromeno[3,4-a]pyrrolizine-11,3'-indoline]-2',6-dione (4d):** 184.4 mg, white solid. TLC: R*_f_* = 0.51 (60% EtOAc/Hexanes); M. P. 210-212 °C. IR (KBr) 3269, 3180, 2860, 1763, 1704, 1619, 1497, 1473, 1351, 1280, 1247, 821, 736; ^1^H NMR (500 MH3.z, DMSO-*d*_6_) *δ* 8.24 (s, 1H), 7.52 (d, *J* = 7.5 Hz, 1H), 7.37 (t, *J* = 7.7 Hz, 1H), 7.18 (t, *J* = 7.5 Hz, 1H), 7.09 (d, *J* = 8.7 Hz, 1H), 6.88 (dd, *J* = 12.4, 8.6 Hz, 2H), 6.27 (s, 1H), 4.72 (s, 1H), 3.87 (d, *J* = 11.1 Hz, 1H), 3.08 (m, 2H), 2.92 (t, *J* = 6.9 Hz, 1H), 2.67 – 2.58 (m, 1H), 1.98 (d, *J* = 6.2 Hz, 1H), 1.92 – 1.83 (m, 1H), 1.82 – 1.79 (m, 1H); ^13^C NMR (126 MHz, DMSO-*d*_6_) *δ* 177.22, 166.94, 149.26, 143.05, 129.86, 128.25, 127.91, 126.91, 125.40, 124.99, 121.68, 118.77, 117.84, 110.33, 76.40, 67.33, 50.73, 47.00, 42.90, 33.26, 26.02; HRMS (ESI+): *m/z* calculated for [C_21_H_17_ClN_2_O_3_ + H]^+^ : 381.10059; found: 381.10350.

**2-bromo-6a,6b,7,8,9,11a-hexahydro-6H-spiro[chromeno[3,4-a]pyrrolizine-11,3'-indoline]-2',6-dione (4e):** 208.4 mg, white solid. TLC: R*_f_* = 0.50 (60% EtOAc/Hexanes); M. P. 205-207 °C. IR (KBr) 3444, 3159, 2128, 1753, 1715, 1620, 1478, 1248, 1145, 886; ^1^H NMR (500 MHz, DMSO-*d*_6_) *δ* 10.16 (s, 1H), 7.77 (d, *J* = 7.1 Hz, 1H), 7.34 (d, *J* = 2.8 Hz, 2H), 7.15 (t, *J* = 7.5 Hz, 1H), 6.98 (d, *J* = 8.6 Hz, 1H), 6.76 (d, *J* = 7.7 Hz, 1H), 6.37 (s, 1H), 4.38 (s, 1H), 4.17 (d, *J* = 11.3 Hz, 1H), 3.39 (s, 1H), 3.15 (s, 1H), 2.63 (s, 1H), 2.37 (s, 2H), 1.86 (d, *J* = 37.4 Hz, 2H), 1.67 (s, 1H); ^13^C NMR (126 MHz, DMSO-*d*_6_) *δ* 177.30, 167.12, 150.17, 143.31, 131.30, 130.18, 129.93, 126.81, 125.68, 121.83, 120.67, 118.77, 114.84, 109.90, 76.58, 67.28, 50.39, 46.59, 42.17, 32.77, 26.01; HRMS (ESI+): *m/z* calculated for [C_21_H_17_BrN_2_O_3_ + H]^+^ : 425.05007; found: 425.05282.

**1'-methyl-6a,6b,7,8,9,11a-hexahydro-6H-spiro[chromeno[3,4-a]pyrrolizine-11,3'-indoline]-2',6-dione (4f):** 162 mg, grayscale white solid. TLC: R*_f_* = 0.40 (55% EtOAc/Hexanes); M. P. 190-192 °C. IR (KBr) 3093, 3050, 3018, 1979, 1739, 1610, 1583, 1468, 1374, 1316, 1235, 707; ^1^H NMR (500 MHz, DMSO-*d*_6_) *δ* 7.83 (d, *J* = 7.3 Hz, 1H), 7.42 (t, *J* = 7.6 Hz, 1H), 7.21 (t, *J* = 7.5 Hz, 1H), 7.15 (t, *J* = 7.7 Hz, 1H), 6.97 (d, *J* = 8.1 Hz, 1H), 6.91 (d, *J* = 7.7 Hz, 1H), 6.76 (t, *J* = 7.4 Hz, 1H), 6.28 (d, *J* = 7.5 Hz, 1H), 4.40 (s, 1H), 4.21 (d, *J* = 11.2 Hz, 1H), 3.41 (d, *J* = 11.0 Hz, 1H), 3.17 (d, *J* = 11.5 Hz, 1H), 2.74 (s, 3H), 2.58 (t, *J* = 6.9 Hz, 1H), 2.36 (dd, *J* = 18.7, 8.2 Hz, 1H), 1.94 – 1.79 (m, 2H), 1.71 – 1.59 (m, 1H); ^13^C NMR (126 MHz, DMSO-*d*_6_) *δ* 175.54, 167.67, 150.75, 144.78, 130.14, 128.67, 127.38, 126.43, 125.38, 123.43, 122.39, 117.70, 116.58, 108.79, 76.16, 67.11, 50.44, 47.04, 42.30, 32.81, 26.06, 25.35; HRMS (ESI+): *m/z* calculated for [C_22_H_20_N_2_O_3_ + H]^+^ : 361.15521; found: 361.15726.

**1'-allyl-6a,6b,7,8,9,11a-hexahydro-6H-spiro[chromeno[3,4-a]pyrrolizine-11,3'-indoline]-2',6-dione (4g):** 179.7 mg, pale yellow solid. TLC: R*_f_* = 0.41 (55% EtOAc/Hexanes); M. P. 208-210 °C. IR (KBr) 3028, 2972, 1959, 1763, 1710, 1643, 1613, 1467, 1336, 959, 727; ^1^H NMR (500 MHz, DMSO-*d*_6_) *δ* 7.85 (d, *J* = 7.3 Hz, 1H), 7.38 (t, *J* = 7.6 Hz, 1H), 7.23 – 7.14 (m, 2H), 6.96 (d, *J* = 8.0 Hz, 1H), 6.78 (dd, *J* = 12.5, 7.4 Hz, 2H), 6.29 (d, *J* = 7.5 Hz, 1H), 5.34 – 5.24 (m, 1H), 4.65 (d, *J* = 10.4 Hz, 1H), 4.42 (s, 1H), 4.21 (d, *J* = 11.1 Hz, 1H), 4.07 (d, *J* = 17.8 Hz, 2H), 3.89 – 3.83 (m, 1H), 3.42 (d, *J* = 11.2 Hz, 1H), 3.22 (s, 1H), 2.62 (t, *J* = 6.7 Hz, 1H), 2.38 (dd, *J* = 19.0, 7.9 Hz, 1H), 1.94 – 1.79 (m, 2H), 1.72 – 1.61 (m, 1H); ^13^C NMR (126 MHz, DMSO-*d*_6_) *δ* 175.45, 167.62, 150.87, 143.81, 130.76, 130.02, 128.70, 127.56, 126.63, 125.32, 123.43, 122.36, 117.65, 116.61, 115.36, 109.42, 76.19, 67.34, 50.45, 47.00, 42.68, 4.71, 32.89, 26.18; HRMS (ESI+): *m/z* calculated for [C_24_H_22_N_2_O_3_ + H]^+^ : 387.17086; found: 387.17336.

**1'-(prop-2-yn-1-yl)-6a,6b,7,8,9,11a-hexahydro-6H-spiro[chromeno[3,4-a]pyrrolizine-11,3'-indoline]-2',6-dione (4h):** 171.1 mg, yellow solid. TLC: R*_f_* = 0.41 (50% EtOAc/Hexanes); M. P. 230-232 °C. IR (KBr) 3060, 2122, 1969, 114, 1611, 1586, 1467, 1380, 1352, 870; ^1^H NMR (500 MHz, DMSO-*d*_6_) *δ* 7.86 (d, *J* = 7.2 Hz, 1H), 7.45 (t, *J* = 7.5 Hz, 1H), 7.25 (t, *J* = 7.2 Hz, 1H), 7.14 (t, *J* = 7.5 Hz, 1H), 6.97 (dd, *J* = 19.9, 7.8 Hz, 2H), 6.74 (t, *J* = 7.2 Hz, 1H), 6.25 (d, *J* = 7.4 Hz, 1H), 4.40 (s, 1H), 4.26 – 4.16 (m, 2H), 4.10 (d, *J* = 17.8 Hz, 1H), 3.43 (d, *J* = 10.0 Hz, 1H), 3.19 (d, *J* = 13.1 Hz, 1H), 3.01 (s, 1H), 2.60 (s, 1H), 2.37 (d, *J* = 9.7 Hz, 1H), 1.87 (dd, *J* = 20.0, 8.8 Hz, 2H), 1.66 (d, *J* = 8.9 Hz, 1H); ^13^C NMR (126 MHz, DMSO-*d*_6_) *δ* 174.79, 167.74, 150.70, 142.89, 130.10, 128.75, 127.40, 126.72, 125.38, 123.54, 122.81, 117.42, 116.58, 109.56, 77.35, 76.28, 74.08, 67.30, 50.45, 46.99, 42.61, 32.84, 28.11, 26.12; HRMS (ESI+): *m/z* calculated for [C_24_H_20_N_2_O_3_ + H]^+^ : 385.15521; found: 385.15782.

**1'-benzyl-6a,6b,7,8,9,11a-hexahydro-6H-spiro[chromeno[3,4-a]pyrrolizine-11,3'-indoline]-2',6-dione (4i):** 198.6 mg, pale yellow solid. TLC: R*_f_* = 0.58 (55% EtOAc/Hexanes); M. P. 212-215 °C. IR (KBr) 3063, 2973, 2524, 1772, 1713, 1610, 1466, 1385, 1302, 1264, 752; ^1^H NMR (500 MHz, DMSO-*d*_6_) *δ* 7.87 (d, *J* = 7.3 Hz, 1H), 7.35 - 7.25(m, 2H), 7.17 (t, *J* = 7.4 Hz, 1H), 7.11 (t, *J* = 7.1 Hz, 1H), 7.04 -7.01 (m, 3H), 6.82 (t, *J* = 7.4 Hz, 1H), 6.62 (d, *J* = 7.7 Hz, 1H), 6.38 - 6.33 (m, 3H), 4.82 (d, *J* = 16.3 Hz, 1H), 4.46 (s, 1H), 4.39 (d, *J* = 16.2 Hz, 1H), 4.29 (d, *J* = 11.2 Hz, 1H), 3.46 (d, *J* = 11.2 Hz, 1H), 3.24 (s, 1H), 2.62 (t, *J* = 6.7 Hz, 1H), 2.40 (dd, *J* = 18.4, 8.1 Hz, 1H), 1.95 – 1.81 (m, 2H), 1.67 (d, *J* = 9.6 Hz, 1H); ^13^C NMR (126 MHz, DMSO-*d*_6_) *δ* 175.85, 167.64, 150.95, 143.61, 135.29, 130.00, 128.93, 128.45, 127.83, 126.91, 126.71, 126.07, 125.35, 123.66, 122.45, 117.61, 116.72, 109.47, 76.18, 67.25, 50.49, 47.08, 42.49, 41.96, 32.92, 26.16; HRMS (ESI+): *m/z* calculated for [C_28_H_24_N_2_O_3_ + H]^+^ : 437.18651; found: 437.18980.

**1'-methyl-6a,6b,7,11a-tetrahydro-6H,9H-spiro[chromeno[3',4':3,4]pyrrolo[1,2-c]thiazole-11,3'-indoline]-2',6-dione (4j):** 155.2 mg, pale yellow solid. TLC: R*_f_* = 0.59 (60% EtOAc/Hexanes); M. P. 182-183 °C. IR (KBr) 3050, 2919, 1958, 1848, 1607, 1465, 1377, 1361, 1196, 883; ^1^H NMR (500 MHz, DMSO-*d*_6_) *δ* 7.70 – 7.66 (m, 1H), 7.48 – 7.43 (m, 1H), 7.24 (t, *J* = 7.4 Hz, 2H), 7.05 – 6.95 (m, 2H), 6.84 (t, *J* = 7.4 Hz, 1H), 6.17 (d, *J* = 7.4 Hz, 1H), 4.48 (t, *J* = 7.3 Hz, 1H), 4.10 (d, *J* = 9.3 Hz, 1H), 3.97 (d, *J* = 10.2 Hz, 1H), 3.76 (dd, *J* = 18.6, 9.8 Hz, 2H), 3.02 – 2.95 (m, 1H), 2.69 (s, 3H); ^13^C NMR (126 MHz, DMSO-*d*_6_) *δ* 175.46, 166.01, 151.22, 144.45, 130.59, 129.38, 127.20, 125.78, 124.88, 123.63, 122.76, 117.79, 116.63, 108.88, 76.01, 70.36, 55.04, 46.45, 44.30, 37.05, 25.52; HRMS (ESI+): *m/z* calculated for [C_21_H_18_N_2_O_3_S + H]^+^ : 379.11163; found: 379.11420.

**1'-allyl-6a,6b,7,11a-tetrahydro-6H,9H-spiro[chromeno[3',4':3,4]pyrrolo[1,2-c]thiazole-11,3'-indoline]-2',6-dione (4k):** 175.9 mg, pale yellow solid. TLC: R*_f_* = 0.61 (60% EtOAc/Hexanes); M. P. 178-181 °C. IR (KBr) 3077, 2560, 2095, 1840, 1641, 1605, 1461, 1366, 1227, 891; ^1^H NMR (500 MHz, DMSO-*d*_6_) *δ* 7.73 - 7.70 (m, 1H), 7.44 – 7.39 (m, 1H), 7.24 (s, 2H), 7.01 (d, *J* = 8.1 Hz, 1H), 6.90 – 6.82 (m, 7.6 Hz, 2H), 6.22 (d, *J* = 7.5 Hz, 1H), 5.23 – 5.14 (m, 1H), 4.78 (d, *J* = 10.3 Hz, 1H), 4.49 (dd, *J* = 18.8, 12.1 Hz, 2H), 4.10 (d, *J* = 9.3 Hz, 1H), 3.99 (t, *J* = 13.3 Hz, 2H), 3.82 (dd, *J* = 25.9, 8.3 Hz, 3H), 3.06 – 2.99 (m, 1H); ^13^C NMR (126 MHz, DMSO-*d*_6_) *δ* 175.39, 166.06, 151.33, 143.60, 131.00, 130.55, 129.44, 127.60, 126.14, 126.03, 124.75, 123.77, 122.77, 117.74, 116.72, 116.29, 109.54, 76.05, 70.33, 55.13, 46.60, 44.59, 41.15, 37.30; HRMS (ESI+): *m/z* calculated for [C_23_H_20_N_2_O_3_S + H]^+^ : 405.12729; found: 405.13086.

**1'-(prop-2-yn-1-yl)-6a,6b,7,11a-tetrahydro-6H,9H-spiro[chromeno[3',4':3,4]pyrrolo[1,2-c]thiazole-11,3'-indoline]-2',6-dione (4l):** 171 mg, pale yellow solid. TLC: R*_f_* = 0.65 (60% EtOAc/Hexanes); M. P. 190-193 °C. IR (KBr) 3011, 2581, 2129, 1719, 1621, 1555, 1466, 1339, 117, 884; ^1^H NMR (500 MHz, DMSO-*d*_6_) *δ* 7.74 – 7.69 (m, 1H), 7.52 – 7.45 (m, 1H), 7.28 (t, *J* = 7.3 Hz, 1H), 7.21 (t, *J* = 7.7 Hz, 1H), 7.06 – 7.03 (m, 1H), 7.00 (d, *J* = 8.1 Hz, 1H), 6.82 (t, *J* = 7.3 Hz, 1H), 6.17 (d, *J* = 7.5 Hz, 1H), 4.48 (t, *J* = 7.2 Hz, 1H), 4.21 (d, *J* = 8.9 Hz, 2H), 4.02 (d, *J* = 8.9 Hz, 2H), 3.98 (d, *J* = 10.3 Hz, 1H), 3.86 – 3.77 (m, 3H), 3.72 (d, *J* = 10.1 Hz, 1H), 3.39 – 3.33 (m, 2H), 3.10 – 3.05 (m, 2H), 2.83 – 2.78 (m, 2H); ^13^C NMR (126 MHz, DMSO-*d*_6_) *δ* 180.51, 171.18, 156.45, 148.72, 136.12, 135.67, 134.56, 132.72, 131.26, 131.15, 129.87, 128.89, 127.89, 122.86, 121.84, 121.42, 114.66, 81.17, 75.45, 60.25, 51.73, 49.71, 46.27, 42.42; HRMS (ESI+): *m/z* calculated for [C_23_H_18_N_2_O_3_S + H]^+^ : 403.11163; found: 403.11507.

**1'-benzyl-6a,6b,7,11a-tetrahydro-6H,9H-spiro[chromeno[3',4':3,4]pyrrolo[1,2-c]thiazole-11,3'-indoline]-2',6-dione (4m):** 200 mg, pale yellow solid. TLC: R*_f_* = 0.57 (55% EtOAc/Hexanes); M. P. 195-197 °C. IR (KBr) 3085, 3053, 1958, 11, 112, 160, 1468, 1365, 1244, 1176, 891; ^1^H NMR (500 MHz, DMSO-*d*_6_) *δ* 7.76 – 7.70 (m, 1H), 7.41 – 7.30 (m, 2H), 7.21 (t, *J* = 7.1 Hz, 1H), 7.13 (d, *J* = 6.8 Hz, 1H), 7.11 – 7.06 (m, 3H), 6.88 (t, *J* = 7.0 Hz, 1H), 6.72 (d, *J* = 7.6 Hz, 1H), 6.49 (d, *J* = 6.9 Hz, 2H), 6.26 (d, *J* = 7.5 Hz, 1H), 4.75 (d, *J* = 16.0 Hz, 1H), 4.53 (t, *J* = 6.8 Hz, 1H), 4.36 (d, *J* = 15.8 Hz, 1H), 4.17 (d, *J* = 9.0 Hz, 1H), 3.99 (d, *J* = 10.1 Hz, 1H), 3.81 (dd, *J* = 16.5, 9.9 Hz, 2H), 3.43 – 3.36 (m, 1H), 3.09 – 3.03 (m, 1H); ^13^C NMR (126 MHz, DMSO-*d*_6_) *δ* 175.77, 165.99, 151.29, 143.46, 135.41, 130.44, 129.49, 128.39, 127.79, 126.99, 126.41, 126.16, 124.54, 123.95, 122.70, 117.59, 116.73, 109.46, 75.76, 70.08, 55.07, 46.06, 44.69, 42.37, 37.22; HRMS (ESI+): *m/z* calculated for [C_27_H_22_N_2_O_3_S + H]^+^ : 455.14293; found: 455.14652.

**1'-benzyl-2-methyl-6a,6b,7,11a-tetrahydro-6H,9H-spiro[chromeno[3',4':3,4]pyrrolo[1,2-c]thiazole-11,3'-indoline]-2',6-dione (4n):** 199.1 mg, pale yellow solid. TLC: R*_f_* = 0.58 (55% EtOAc/Hexanes); M. P. 190-193 °C. IR (KBr) 3085, 195, 1751, 1718, 1607, 1467, 1350, 1155, 980; ^1^H NMR (500 MHz, DMSO-*d*_6_) *δ* 7.73 – 7.68 (m, 1H), 7.37 – 7.32 (m, 1H), 7.19 (dt, *J* = 15.6, 7.4 Hz, 3H), 7.12 – 7.06 (m, 2H), 6.97 (d, *J* = 8.1 Hz, 1H), 6.74 (d, *J* = 7.7 Hz, 1H), 6.50 (d, *J* = 7.3 Hz, 2H), 5.98 (s, 1H), 4.78 (d, *J* = 16.0 Hz, 1H), 4.53 (t, *J* = 7.0 Hz, 1H), 4.34 (d, *J* = 15.9 Hz, 1H), 4.10 (d, *J* = 9.2 Hz, 1H), 3.99 (d, *J* = 10.3 Hz, 1H), 3.79 (t, *J* = 8.5 Hz, 2H), 3.07 – 3.01 (m, 1H), 1.90 (s, 3H); ^13^C NMR (126 MHz, DMSO-*d*_6_) *δ* 181.01, 171.51, 154.47, 148.82, 140.72, 138.02, 135.64, 135.23, 133.60, 133.12, 132.29, 131.63, 131.63, 129.95, 127.97, 122.58, 121.71, 114.64, 80.97, 75.38, 60.30, 51.49, 49.85, 47.71, 42.46, 25.36; HRMS (ESI+): *m/z* calculated for [C_28_H_25_N_2_O_3_S + H]^+^ : 469.15859; found: 469.16256.

**3-(4-methoxyphenyl)-2,3,3a,9b-tetrahydro-4H-spiro[chromeno[3,4-c]pyrrole-1,3'-indoline]-2',4-dione (7a):** 179.3 mg, white solid. TLC: R*_f_* = 0.47 (55% EtOAc/Hexanes); M. P. 193-195 °C. IR (KBr) 3337, 3093, 2069, 1767, 1716, 1618, 1565, 1470, 1396, 1222, 1196, 865; ^1^H NMR (500 MHz, DMSO-*d*_6_) *δ* 7.75 (s, 1H), 7.69 (d, *J* = 7.7 Hz, 2H), 7.62 (d, *J* = 7.3 Hz, 1H), 7.33 (t, *J* = 7.7 Hz, 1H), 7.21 (t, *J* = 7.4 Hz, 1H), 7.15 (t, *J* = 7.7 Hz, 1H), 6.95 (d, *J* = 7.8 Hz, 3H), 6.82 – 6.75 (m, 2H), 6.31 (d, *J* = 7.6 Hz, 1H), 5.64 (s, 1H), 3.89 (d, *J* = 10.6 Hz, 1H), 3.83 (s, 3H), 3.30 (d, *J* = 10.7 Hz, 1H); ^13^C NMR (126 MHz, DMSO-*d*_6_) *δ* 178.58, 166.54, 157.32, 149.47, 141.41, 135.71, 128.17, 127.63, 126.54, 122.60, 122.33, 120.96, 115.93, 115.24, 112.39, 108.60, 72.16, 60.75, 53.85, 47.14, 45.8; HRMS (ESI+): *m/z* calculated for [C_25_H_20_N_2_O_4_ + H]^+^ : 413.15013; found: 413.15295.

**3-(4-methoxyphenyl)-1'-methyl-2,3,3a,9b-tetrahydro-4H-spiro[chromeno[3,4-c]pyrrole-1,3'-indoline]-2',4-dione (7b):** 196.2 mg, white solid. TLC: R*_f_* = 0.50 (55% EtOAc/Hexanes); M. P. 200-202 °C. IR (KBr) 3151, 2971, 1733, 1694, 1613, 1471, 1376, 1309, 1176, 830; ^1^H NMR (500 MHz, DMSO-*d*_6_) *δ* 9.02 (s, 1H), 7.64 (d, *J* = 7.5 Hz, 2H), 7.49 (d, *J* = 7.2 Hz, 1H), 7.12 (t, *J* = 7.4 Hz, 1H), 7.01 (t, *J* = 7.3 Hz, 1H), 6.90 - 6.81 (m, 4H), 6.59 (t, *J* = 7.2 Hz, 1H), 6.54 (d, *J* = 7.8 Hz, 1H), 6.33 (d, *J* = 7.1 Hz, 1H), 5.34 (t, *J* = 7.7 Hz, 1H), 4.32 – 4.20 (m, 2H), 3.25 (s, 3H), 3.15 (s, 3H); ^13^C NMR (126 MHz, DMSO-*d*_6_) *δ* 180.20, 171.31, 158.14, 155.12, 143.75, 137.05, 128.97, 128.66, 128.59, 128.48, 127.84, 125.16, 124.00, 121.23, 118.19, 114.72, 113.25, 107.67, 71.20, 62.69, 56.22, 55.00, 51.26, 46.76, 25.98; HRMS (ESI+): *m/z* calculated for [C_26_H_22_N_2_O_4_ + H]^+^ : 427.16578; found: 427.16931.

**3-(4-methoxyphenyl)-1'-(prop-2-yn-1-yl)-2,3,3a,9b-tetrahydro-4H-spiro[chromeno[3,4-c]pyrrole-1,3'-indoline]-2',4-dione (7c):** 191.4 mg, pale yellow solid. TLC: R*_f_* = 0.45 (50% EtOAc/Hexanes); M. P. 180-182 °C. IR (KBr) 3049, 2126, 1755, 1619, 1610, 1482, 1306, 1224, 1180, 813; ^1^H NMR (500 MHz, DMSO-*d*_6_) *δ* 7.73 (d, *J* = 7.2 Hz, 1H), 7.60 (d, *J* = 7.6 Hz, 2H), 7.41 (t, *J* = 7.6 Hz, 1H), 7.25 (t, *J* = 7.3 Hz, 1H), 7.17 (t, *J* = 7.7 Hz, 1H), 7.00 - 6.94 (m, 4H), 6.76 (t, *J* = 7.4 Hz, 1H), 6.16 (d, *J* = 7.5 Hz, 1H), 5.29 (s, 1H), 4.12 (s, 2H), 4.07 (d, *J* = 5.8 Hz, 1H), 4.03 (d, *J* = 11.0 Hz, 1H), 3.76 (s, 3H), 3.54 (d, *J* = 8.8 Hz, 1H), 2.96 (s, 1H); ^13^C NMR (126 MHz, DMSO-*d*_6_) *δ* 176.96, 168.06, 158.48, 150.46, 142.29, 137.14, 129.58, 129.13, 1278.47, 128.13, 127.39, 124.17, 123.63, 123.22, 117.02, 116.53, 113.74, 109.28, 73.92, 77.36, 73.20, 62.17, 55.20, 47.88, 47.06, 28.08; HRMS (ESI+): *m/z* calculated for [C_28_H_22_N_2_O_4_ + H]^+^ : 451.16578; found: 451.16956.

**3-(4-fluorophenyl)-2,3,3a,9b-tetrahydro-4H-spiro[chromeno[3,4-c]pyrrole-1,3'-indoline]-2',4-dione (7d):** 182.2 mg, white solid. TLC: R*_f_* = 0.45 (50% EtOAc/Hexanes); M. P. 186-188 °C. IR (KBr) 3329, 2928, 2138, 1765, 1620, 1492, 1332, 1286, 1240, 1159, 815; ^1^H NMR (500 MHz, DMSO-*d*_6_) *δ* 10.05 (s, 1H), 7.72 (t, *J* = 6.5 Hz, 2H), 7.65 (d, *J* = 7.3 Hz, 1H), 7.30 – 7.19 (m, 4H), 7.14 (t, *J* = 7.4 Hz, 1H), 7.00 (d, *J* = 8.1 Hz, 1H), 6.83 (t, *J* = 7.4 Hz, 1H), 6.71 (d, *J* = 7.6 Hz, 1H), 6.27 (d, *J* = 7.5 Hz, 1H), 5.33 (s, 1H), 4.14 (d, *J* = 5.5 Hz, 1H), 3.98 (t, *J* = 12.4 Hz, 1H), 3.54 (dd, *J* = 11.0, 2.8 Hz, 1H); ^13^C NMR (126 MHz, DMSO-*d*_6_) *δ* 179.83, 168.02, 162.34, 160.42, 150.64, 142.71, 141.55, 129.53, 129.16, 128.88, 127.56, 124.37, 123.65, 122.16, 117.55, 116.58, 115.08, 109.63, 73.17, 61.58, 47.80, 46.44; ^19^F NMR: (471 MHz, DMSO-d6) δ 111.44; HRMS (ESI+): *m/z* calculated for [C_25_H_20_N_2_O_4_ + H]^+^ : 413.15013; found: 413.15226.

**1'-allyl-3-(4-fluorophenyl)-2,3,3a,9b-tetrahydro-4H-spiro[chromeno[3,4-c]pyrrole-1,3'-indoline]-2',4-dione (7e):** 193.8 mg, white solid. TLC: R*_f_* = 0.50 (40% EtOAc/Hexanes); M. P. 192-194 °C. IR (KBr) 3065, 3078, 2031, 1707, 1641, 1615, 1602, 1468, 1331, 1275, 1260, 1239, 1183, 810; ^1^H NMR (500 MHz, DMSO-*d*_6_) *δ* 7.76 – 7.71 (m, 3H), 7.35 (t, *J* = 7.6 Hz, 1H), 7.27 – 7.18 (m, 4H), 6.98 (d, *J* = 8.1 Hz, 1H), 6.84 – 6.76 (m, 2H), 6.23 (d, *J* = 7.4 Hz, 1H), 5.37 (s, 1H), 5.23 – 5.14 (m, 1H), 4.72 (d, *J* = 10.3 Hz, 1H), 4.33 (d, *J* = 17.2 Hz, 1H), 4.18 (d, *J* = 5.8 Hz, 1H), 4.07 (dd, *J* = 24.4, 13.7 Hz, 2H), 3.81 (dd, *J* = 16.6, 4.0 Hz, 1H), 3.58 (d, *J* = 10.8 Hz, 1H); ^13^C NMR (126 MHz, DMSO-*d*_6_) *δ* 177.69, 168.04, 162.49, 160.48, 150.68, 143.26, 141.52, 131.17, 129.62, 128.86, 128.39, 127.71, 124.20, 123.67, 122.90, 117.25 116.70, 115.91, 115.17, 109.20, 73.08, 61.74, 47.87, 46.75, 40.95; ^19^F NMR: (471 MHz, DMSO-d6) δ 116.11; HRMS (ESI+): *m/z* calculated for [C_27_H_21_FN_2_O_3_ + H]^+^ : 441.16144; found: 441.16503.

**1'-benzyl-3-(4-fluorophenyl)-2,3,3a,9b-tetrahydro-4H-spiro[chromeno[3,4-c]pyrrole-1,3'-indoline]-2',4-dione (7f):** 233 mg, white solid. TLC: R*_f_* = 0.60 (40% EtOAc/Hexanes); M. P. 192-195 °C. IR (KBr) 3039, 2201, 1745, 1607, 1568, 1494, 1466, 1309, 1239, 950, 733; ^1^H NMR (500 MHz, DMSO-*d*_6_) *δ* 7.29 – 7.72 (m, 3H), 7.37 (t, *J* = 7.6 Hz, 1H), 7.29 – 7.17 (m, 4H), 7.14 (t, *J* = 7.0 Hz, 1H), 7.10 – 7.04 (m, 3H), 6.85 (t, *J* = 7.3 Hz, 1H), 6.60 (d, *J* = 7.5 Hz, 1H), 6.45 (d, *J* = 7.2 Hz, 2H), 6.30 (d, *J* = 7.5 Hz, 1H), 5.39 (s, 1H), 4.85 (d, *J* = 16.1 Hz, 1H), 4.34 (d, *J* = 16.0 Hz, 1H), 4.26 (d, *J* = 5.7 Hz, 1H), 4.14 (d, *J* = 10.9 Hz, 1H), 3.62 (d, *J* = 11.0 Hz, 1H); ^13^C NMR (126 MHz, DMSO-*d*_6_) *δ* 177.90, 167.95, 162.28, 160.35, 150.64, 143.06, 141.30, 135.43, 129.48, 128.80, 128.38, 128.27, 127.88, 126.87, 126.19, 124.30, 123.81, 122.87, 117.07, 116.70, 115.03, 114.86, 109.09, 72.82, 61.49, 47.81, 46.28, 42.13; ^19^F NMR: (471 MHz, DMSO-d6) δ 111.27; HRMS (ESI+): *m/z* calculated for [C_23_H_20_N_2_O_5_ + H]^+^ : 405.14504; found: 405.14867.

**3-phenyl-2,3,3a,9b-tetrahydro-4H-spiro[chromeno[3,4-c]pyrrole-1,3'-indoline]-2',4-dione (7g):** 185.5 mg, pale yellow solid. TLC: R*_f_* = 0.55 (60% EtOAc/Hexanes); M. P. 202-205 °C. IR (KBr) 3330, 2927, 1968, 1767, 1704, 1621, 1469, 1395, 1195, 867; ^1^H NMR (500 MHz, DMSO-*d*_6_) *δ* 10.03 (s, 1H), 7.72 – 7.63 (m, 3H), 7.44 – 7.37 (m, 2H), 7.28 (s, 2H), 7.21 (t, *J* = 7.6 Hz, 1H), 7.14 (t, *J* = 7.2 Hz, 1H), 7.00 (d, *J* = 8.1 Hz, 1H), 6.83 (t, *J* = 7.1 Hz, 1H), 6.70 (d, *J* = 7.5 Hz, 1H), 6.27 (d, *J* = 7.4 Hz, 1H), 5.33 (s, 1H), 4.08 (d, *J* = 5.5 Hz, 1H), 3.99 (d, *J* = 11.1 Hz, 1H), 3.56 (d, *J* = 11.0 Hz, 1H); ^13^C NMR (126 MHz, DMSO-*d*_6_) *δ* 179.80, 167.96, 150.64, 145.39, 142.68, 129.45, 129.22, 128.99, 128.29, 127.53, 126.99, 124.31, 123.56, 122.10, 117.58, 116.53, 109.56, 73.21, 62.23, 47.82, 46.59; HRMS (ESI+): *m/z* calculated for [C_24_H_18_N_2_O_3_ + H]^+^ : 383.13956; found: 383.14176.

**1'-methyl-3-phenyl-2,3,3a,9b-tetrahydro-4H-spiro[chromeno[3,4-c]pyrrole-1,3'-indoline]-2',4-dione (7h):** 172.4 mg, white solid. TLC: R*_f_* = 0.63 (55% EtOAc/Hexanes); M. P. 190-192 °C. IR (KBr) 3082, 3057, 1984, 1765, 1701, 1610, 146, 1375, 1258, 1197, 853; ^1^H NMR (500 MHz, DMSO-*d*_6_) *δ* 7.73 – 7.68 (m, 3H), 7.43 – 7.36 (m, 3H), 7.29 (t, *J* = 7.1 Hz, 1H), 7.25 – 7.14 (m, 2H), 6.99 (d, *J* = 8.1 Hz, 1H), 6.91 (d, *J* = 7.6 Hz, 1H), 6.78 (t, *J* = 7.3 Hz, 1H), 6.20 (d, *J* = 7.5 Hz, 1H), 5.35 (s, 1H), 4.08 (d, *J* = 5.6 Hz, 1H), 4.03 (d, *J* = 11.0 Hz, 1H), 2.72 (s, 3H); ^13^C NMR (126 MHz, DMSO-*d*_6_) *δ* 177.77, 167.96, 150.52, 145.30, 144.16, 129.66, 129.07, 128.52, 128.36, 127.37, 127.02, 123.98, 123.48, 122.88, 117.25, 116.59, 108.52, 73.14, 62.38, 47.87, 46.69, 25.38; HRMS (ESI+): *m/z* calculated for [C_25_H_20_N_2_O_3_ + H]^+^ : 397.15521; found: 397.15816.

**1'-allyl-3-phenyl-2,3,3a,9b-tetrahydro-4H-spiro[chromeno[3,4-c]pyrrole-1,3'-indoline]-2',4-dione (7i):** 194.3 mg, white solid. TLC: R*_f_* = 0.48 (50% EtOAc/Hexanes); M. P. 201-203 °C. IR (KBr) 3081, 2435, 1710, 1644, 1614, 1467, 1307, 1026, 825; ^1^H NMR (500 MHz, DMSO-*d*_6_) *δ* 7.75 – 7.68 (m, 3H), 7.41 (t, *J* = 7.2 Hz, 2H), 7.35 (t, *J* = 7.5 Hz, 1H), 7.29 (t, *J* = 7.0 Hz, 1H), 7.21 (t, *J* = 7.4 Hz, 2H), 6.98 (d, *J* = 8.0 Hz, 1H), 6.80 (dd, *J* = 11.8, 7.5 Hz, 2H), 6.24 (d, *J* = 7.5 Hz, 1H), 5.37 (s, 1H), 5.25 – 5.14 (m, 1H), 4.72 (d, *J* = 10.3 Hz, 1H), 4.32 (d, *J* = 17.3 Hz, 1H), 4.14 (d, *J* = 6.2 Hz, 1H), 4.09 (s, 1H), 4.05 (d, *J* = 10.9 Hz, 1H), 3.81 (dd, *J* = 16.8, 4.0 Hz, 1H), 3.62 – 3.54 (m, 1H); ^13^C NMR (126 MHz, DMSO-*d*_6_) *δ* 177.66, 167.97, 150.68, 145.35, 143.23, 131.15, 129.54, 129.13, 128.45, 128.37, 127.68, 127.08, 127.01, 124.13, 123.58, 122.84, 117.24, 116.64, 115.87, 109.13, 73.12, 62.39, 47.89, 46.92, 40.92; HRMS (ESI+): *m/z* calculated for [C_27_H_22_N_2_O_3_ + H]^+^ : 423.17086; found: 423.17453.

**3-phenyl-1'-(prop-2-yn-1-yl)-2,3,3a,9b-tetrahydro-4H-spiro[chromeno[3,4-c]pyrrole-1,3'-indoline]-2',4-dione (7j):** 189.2 mg, pale yellow solid. TLC: R*_f_* = 0.52 (50% EtOAc/Hexanes); M. P. 195-198 °C. IR (KBr) 3056, 2120, 1989, 1892, 1854, 1613, 146, 1427, 1304, 1237, 1194, 840; ^1^H NMR (500 MHz, DMSO-*d*_6_) *δ* 7.78 – 7.68 (m, 3H), 7.45 – 7.37 (m, 3H), 7.32 – 7.23 (m, 2H), 7.18 (t, *J* = 7.6 Hz, 1H), 7.01 – 6.94 (t, *J* = 7.9 Hz, 2H), 6.77 (t, *J* = 7.3 Hz, 1H), 6.17 (d, *J* = 7.5 Hz, 1H), 5.34 (s, 1H), 4.20 – 4.09 (m, 3H), 4.04 (d, *J* = 10.9 Hz, 1H), 3.60 (dd, *J* = 10.9, 3.3 Hz, 1H), 2.96 (s, 1H); ^13^C NMR (126 MHz, DMSO-*d*_6_) *δ* 175.29, 166.07, 148.75, 143.40, 140.52, 127.82, 127.38, 126.72, 126.59, 125.74, 125.33, 125.16, 122.25, 121.87, 121.51, 114.94, 114.76, 107.50, 75.15, 71.74, 71.65, 60.76, 46.35, 45.66, 26.45; HRMS (ESI+): *m/z* calculated for [C_27_H_20_N_2_O_3_ + H]^+^ : 421.15521; found: 421.15781.

**1'-benzyl-3-phenyl-2,3,3a,9b-tetrahydro-4H-spiro[chromeno[3,4-c]pyrrole-1,3'-indoline]-2',4-dione (7k):** 207.9 mg, white solid. TLC: R*_f_* = 0.67 (40% EtOAc/Hexanes); M. P. 210-212 °C. IR (KBr) 3030, 2130, 1766, 1734, 1567, 1466, 1366, 1219, 1193, 1180, 897; ^1^H NMR (500 MHz, DMSO-*d*_6_) *δ* 7.77 – 7.71 (m, 3H), 7.43 – 7.37 (m, 2H), 7.34 (t, *J* = 7.4 Hz, 1H), 7.32 – 7.22 (m, 2H), 7.19 (t, *J* = 7.3 Hz, 1H), 7.12 (t, *J* = 7.0 Hz, 1H), 7.09 – 7.02 (m, 3H), 6.82 (t, *J* = 7.3 Hz, 1H), 6.58 (d, *J* = 7.4 Hz, 1H), 6.46 (d, *J* = 7.4 Hz, 2H), 6.33 (d, *J* = 7.6 Hz, 1H), 5.44 (s, 1H), 4.88 (d, *J* = 16.0 Hz, 1H), 4.31 (d, *J* = 15.8 Hz, 1H), 4.11 (d, *J* = 10.8 Hz, 2H), 3.57 (s, 1H); ^13^C NMR (126 MHz, DMSO-*d*_6_) *δ* 176.24, 166.08, 148.99, 143.42, 141.40, 127.70, 127.70, 127.48, 127.48, 126.64, 126.64, 126.51, 126.24, 126.24, 125.23, 125.13, 125.13, 124.46, 124.46, 122.35, 122.35, 122.00, 122.00, 121.12, 121.12, 115.31, 115.31, 114.95, 114.95, 107.36, 107.36, 71.25, 60.45, 46.33, 44.95, 40.62; HRMS (ESI+): *m/z* calculated for [C_31_H_24_N_2_O_3_ + H]^+^ : 473.18651; found: 473.19020.

**8-methyl-3-phenyl-2,3,3a,9b-tetrahydro-4H-spiro[chromeno[3,4-c]pyrrole-1,3'-indoline]-2',4-dione (7l):** 176.4 mg, white solid. TLC: R*_f_* = 0.52 (55% EtOAc/Hexanes); M. P. 220-222 °C. IR (KBr) 3321, 3080, 191, 1764, 104, 1622, 1622, 1470, 1394, 1212, 1198, 879; ^1^H NMR (500 MHz, DMSO-*d*_6_) *δ* 10.00 (s, 1H), 7.73 - 7.67 (m, 2H), 7.65 - 7.61 (m, 1H), 7.39 (t, *J* = 7.2 Hz, 2H), 7.28 (t, *J* = 6.6 Hz, 2H), 7.14 (t, *J* = 7.4 Hz, 1H), 7.03 - 6.97 (m, 1H), 6.86 (d, *J* = 8.2 Hz, 1H), 6.71 (d, *J* = 7.6 Hz, 1H), 6.02 (s, 1H), 5.36 (s, 1H), 4.02 (s, 1H), 3.89 (d, *J* = 11.1 Hz, 1H), 3.47 (d, *J* = 10.7 Hz, 1H), 1.95 (s, 3H); ^13^C NMR (126 MHz, DMSO-*d*_6_) *δ* 177.95, 166.15, 146.75, 143.58, 140.85, 130.45, 127.57, 127.47, 127.45, 126.40, 126.03, 125.07, 122.27, 120.23, 115.26, 114.33, 107.63, 71.44, 60.41, 46.06, 45.08, 18.41; HRMS (ESI+): *m/z* calculated for [C_25_H_20_N_2_O_3_ + H]^+^ : 397.15521; found: 397.15874.

**8-methoxy-3-phenyl-2,3,3a,9b-tetrahydro-4H-spiro[chromeno[3,4-c]pyrrole-1,3'-indoline]-2',4-dione (7m):** 175.3 mg, white solid. TLC: R*_f_* = 0.61 (55% EtOAc/Hexanes); M. P. 210-213 °C. IR (KBr) 3314, 3028, 2938, 2055, 1765, 1702, 1621, 1469, 1392, 1256, 1241, 111, 881; ^1^H NMR (500 MHz, DMSO-*d*_6_) *δ* 10.05 (s, 1H), 7.72 - 7.76 (m, 2H), 7.65 (d, *J* = 7.3 Hz, 1H), 7.43 - 7.37 (m, 2H), 7.29 (s, 2H), 7.15 (t, *J* = 7.4 Hz, 1H), 6.96 – 6.92 (m, 1H), 6.78 (d, *J* = 8.6 Hz, 1H), 6.73 (d, *J* = 7.6 Hz, 1H), 5.72 (s, 1H), 5.34 (s, 1H), 4.09 (d, *J* = 6.0 Hz, 1H), 3.93 (d, *J* = 11.1 Hz, 1H), 3.51 (dd, *J* = 11.1, 3.8 Hz, 1H), 3.35 (s, 3H); ^13^C NMR (126 MHz, DMSO-*d*_6_) *δ* 179.65, 167.98, 154.58, 145.32, 144.37, 142.65, 129.34, 129.17, 128.22, 126.92, 124.23, 122.05, 118.10, 117.24, 114.57, 111.40, 109.49, 73.18, 62.26, 54.75, 47.39, 46.99; HRMS (ESI+): *m/z* calculated for [C_25_H_20_N_2_O_4_ + H]^+^ : 413.15013; found: 413.15226.

**8-bromo-3-phenyl-2,3,3a,9b-tetrahydro-4H-spiro[chromeno[3,4-c]pyrrole-1,3'-indoline]-2',4-dione (7n):** 209.9 mg, yellow solid. TLC: R*_f_* = 0.53 (50% EtOAc/Hexanes); M. P. 208-210 °C. IR (KBr) 3319, 3039, 2120, 155, 1620, 1474, 1371, 1195, 880, 560; ^1^H NMR (500 MHz, DMSO-*d*_6_) *δ* 10.10 (s, 1H), 7.97 (s, 1H), 7.90 (s, 1H), 7.70 -7.65 (m, 3H), 7.43 – 7.37 (m, 2H), 7.30 (d, *J* = 8.9 Hz, 2H), 7.16 (s, 1H), 7.02 – 6.99 (m, 1H), 6.75 (d, *J* = 7.3 Hz, 1H), 6.32 (s, 1H), 5.32 (s, 1H), 4.13 (s, 1H), 4.00 (d, *J* = 11.7 Hz, 1H), 2.36 (s, 1H); ^13^C NMR (126 MHz, DMSO-*d*_6_) *δ* 179.71, 167.53, 150.01, 145.20, 142.62, 131.75, 130.04, 129.70, 128.73, 128.39, 127.12, 127.01, 124.45, 122.33, 120.26, 118.085, 114.97, 109.65, 73.33, 62.35, 47.41, 46.35; HRMS (ESI+): *m/z* calculated for [C_24_H_17_BrN_2_O_3_ + H]^+^ : 461.05008; found: 461. 05356.

**8-chloro-3-phenyl-2,3,3a,9b-tetrahydro-4H-spiro[chromeno[3,4-c]pyrrole-1,3'-indoline]-2',4-dione (7o):** 185.5 mg, white solid. TLC: R*_f_* = 0.51 (50% EtOAc/Hexanes); M. P. 221-224 °C. IR (KBr) 3320, 3042, 2120, 1883, 1621, 1579, 1341, 1428, 1196, 872, 701; ^1^H NMR (500 MHz, DMSO-*d*_6_) *δ* 10.10 (s, 1H), 7.92 (s, 1H), 7.85 (s, 1H), 7.70 – 7.65 (m, 2H), 7.56 (d, *J* = 8.7 Hz, 1H), 7.43 – 7.35 (m, 2H), 7.29 (s, 2H), 7.16 (s, 1H), 7.08 – 7.04 (m, 1H), 6.75 (d, *J* = 7.8 Hz, 1H), 6.18 (s, 1H), 5.32 (s, 1H), 4.14 (s, 1H), 4.00 (d, *J* = 10.8 Hz, 1H), 2.36 (s, 1H); ^13^C NMR (126 MHz, DMSO-*d*_6_) *δ*  179.86, 167.49, 149.45, 145.11, 142.52, 129.59, 129.58, 128.79, 128.26, 126.99, 126.91, 126.89, 124.35, 123.55, 122.20, 118.38, 110.48, 109.55, 73.17, 62.20, 47.27, 46.25; HRMS (ESI+): *m/z* calculated for [C_24_H_17_ClN_2_O_3_ + H]^+^ : 417.10053; found: 417.10690.

**3. Copies of ^1^H NMR and ^13^C NMR Spectra**

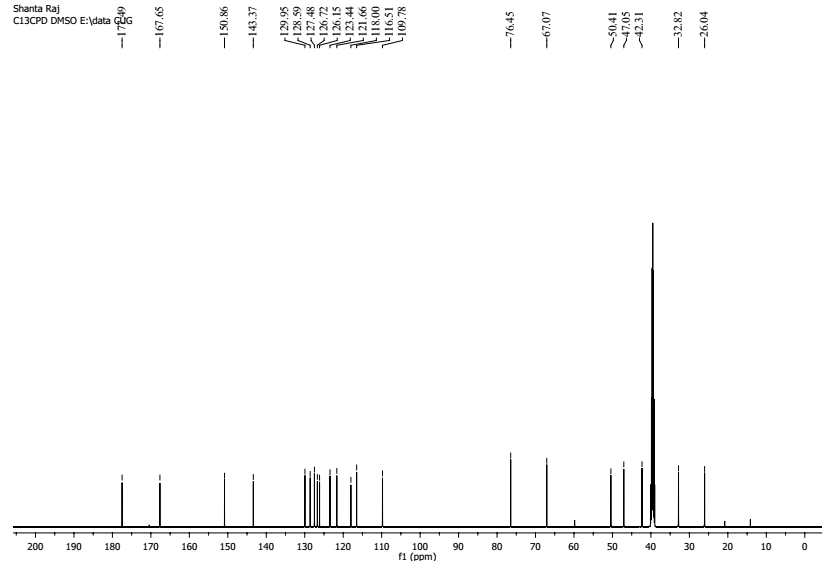


**Figure 3.1** ^1^H NMR (upper) and ^13^C NMR (bottom) of compound **4a**.


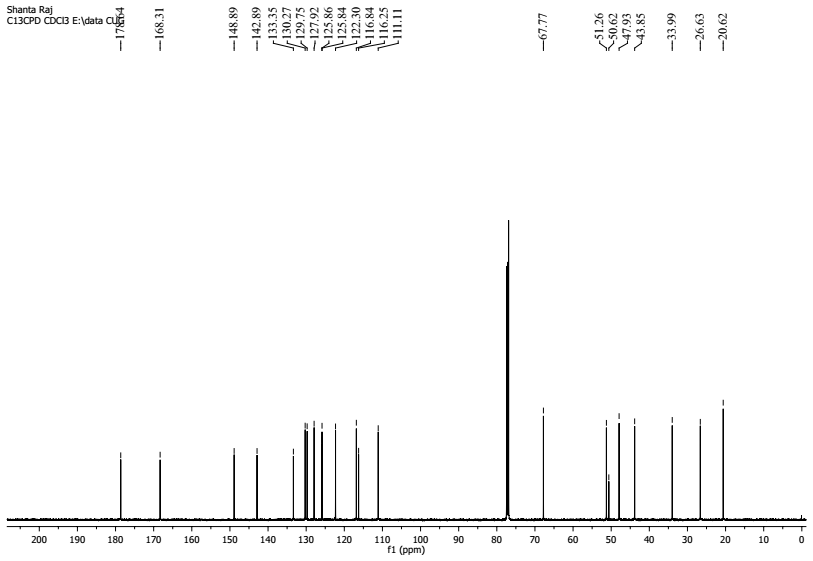


**Figure 3.2** ^1^H NMR (upper) and ^13^C NMR (bottom) of compound **4b**.

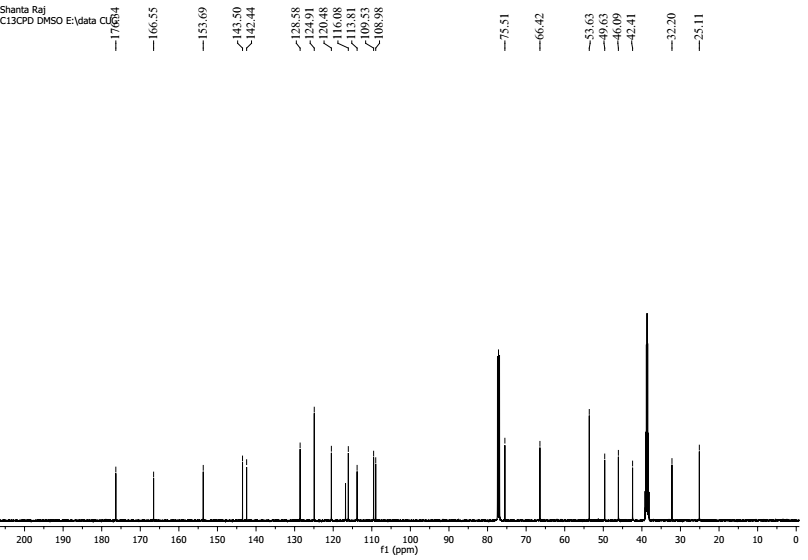


**Figure 3.3** ^1^H NMR (upper) and ^13^C NMR (bottom) of compound **4c**.


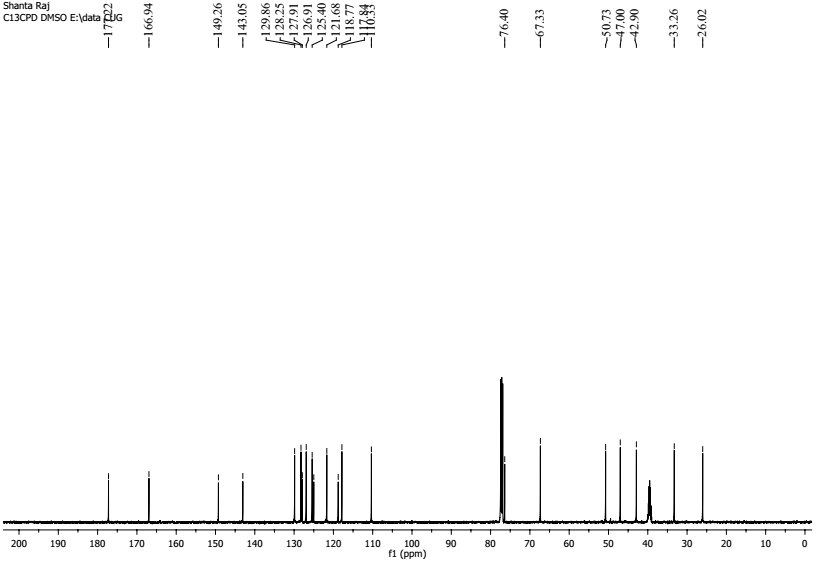


**Figure 3.4** ^1^H NMR (upper) and ^13^C NMR (bottom) of compound **4d**.


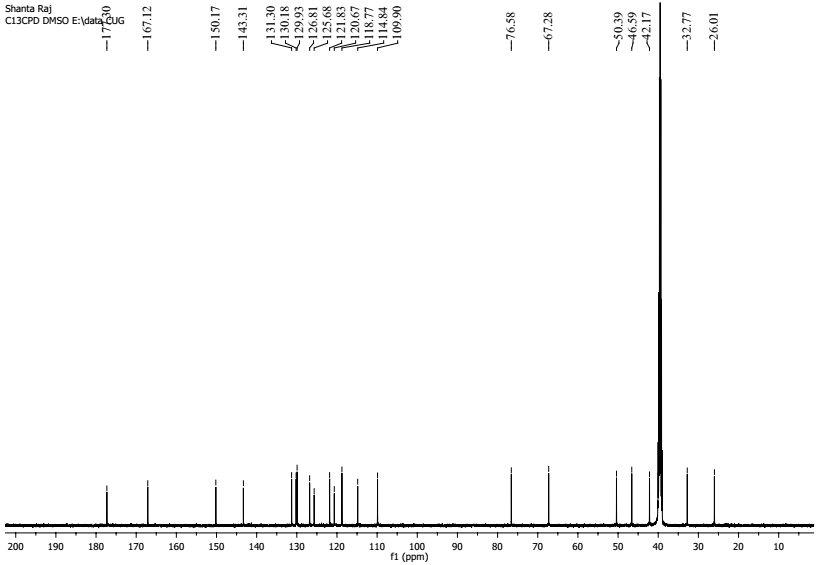


**Figure 3.5** ^1^H NMR (upper) and ^13^C NMR (bottom) of compound **4e**.

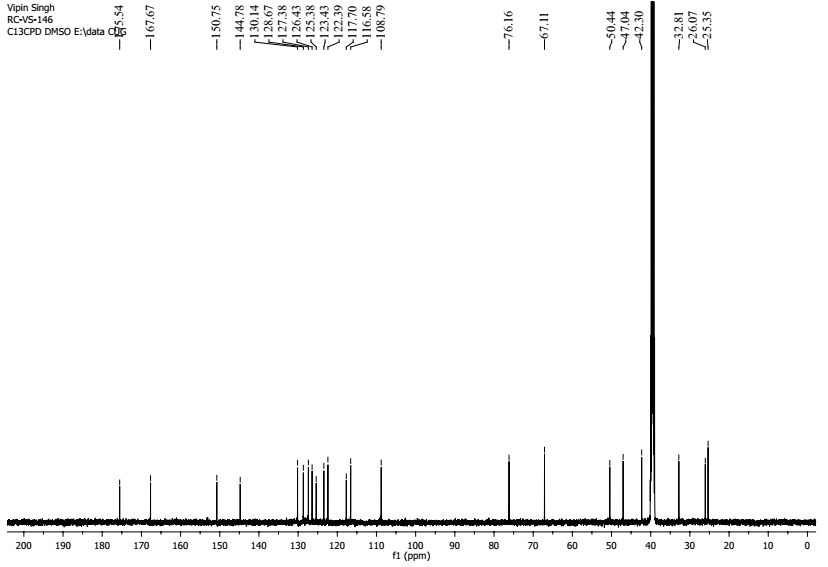


**Figure 3.6** ^1^H NMR (upper) and ^13^C NMR (bottom) of compound **4f**

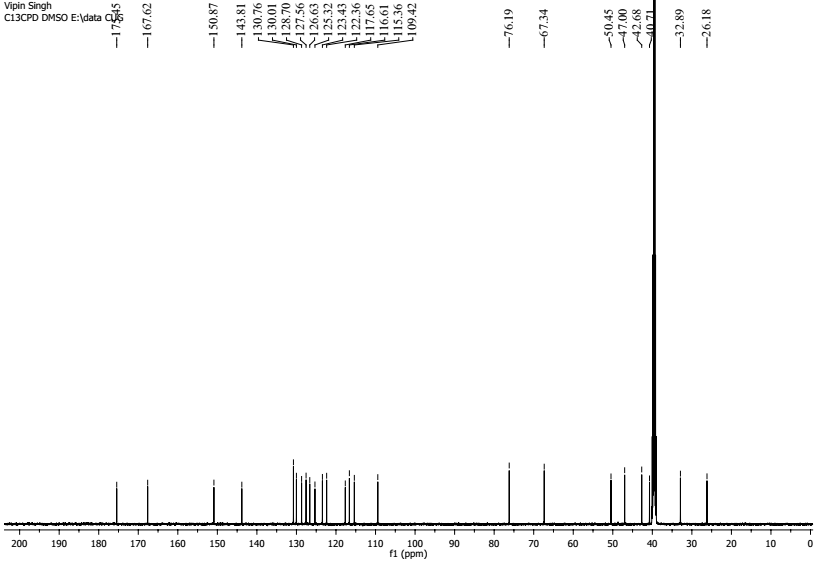


**Figure 3.7** ^1^H NMR (upper) and ^13^C NMR (bottom) of compound **4g**

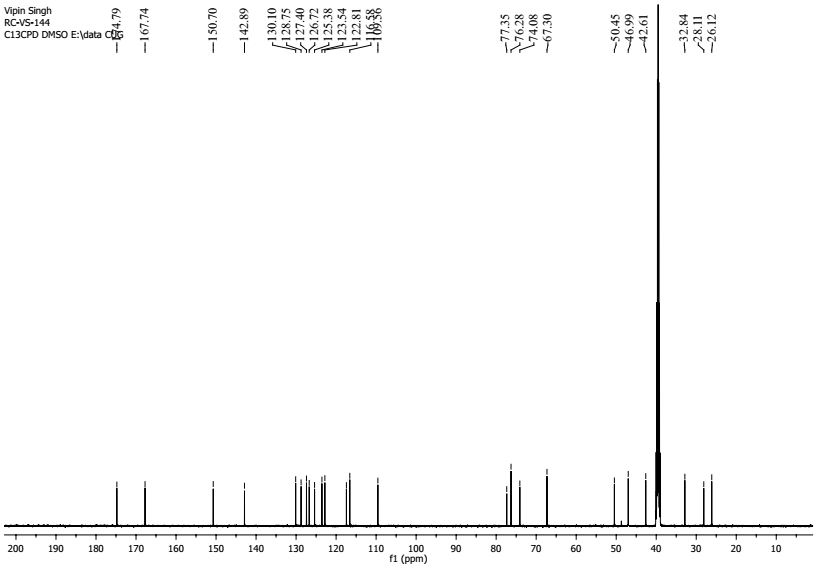


**Figure 3.8** ^1^H NMR (upper) and ^13^C NMR (bottom) of compound **4h**

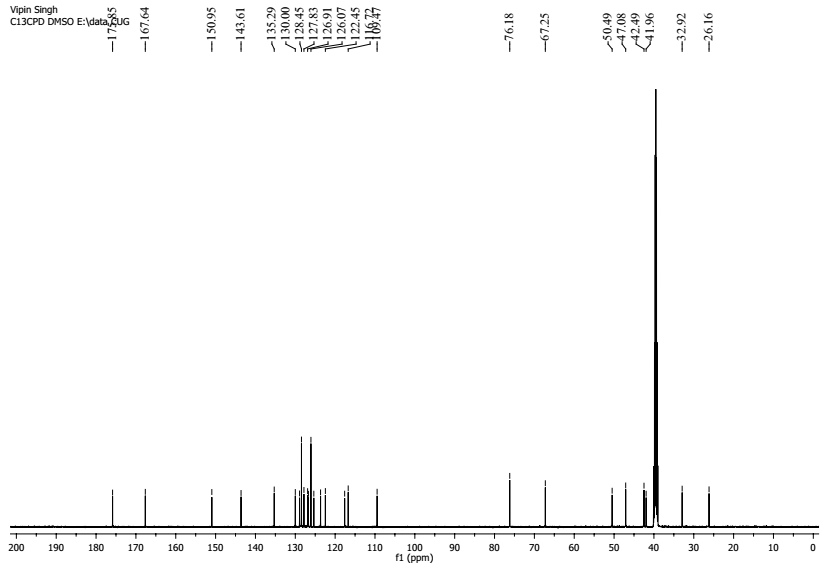


**Figure 3.9** ^1^H NMR (upper) and ^13^C NMR (bottom) of compound **4i**

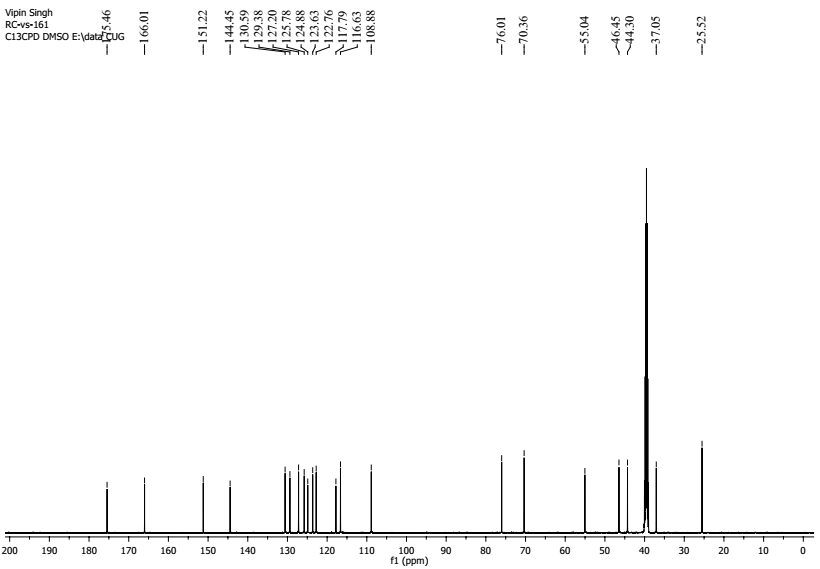


**Figure 3.10** ^1^H NMR (upper) and ^13^C NMR (bottom) of compound **4j**

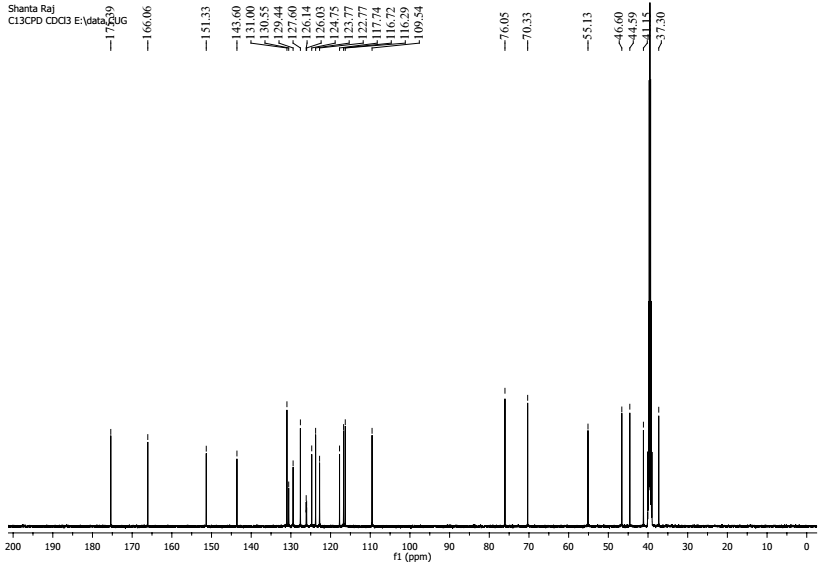


**Figure 3.11** ^1^H NMR (upper) and ^13^C NMR (bottom) of compound **4k**

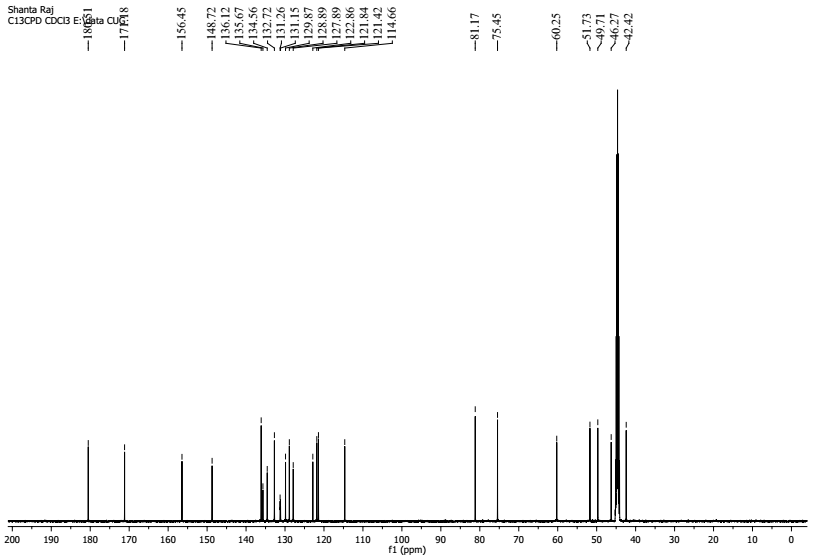
**Figure 3.12** ^1^H NMR (upper) and ^13^C NMR (bottom) of compound **4l**

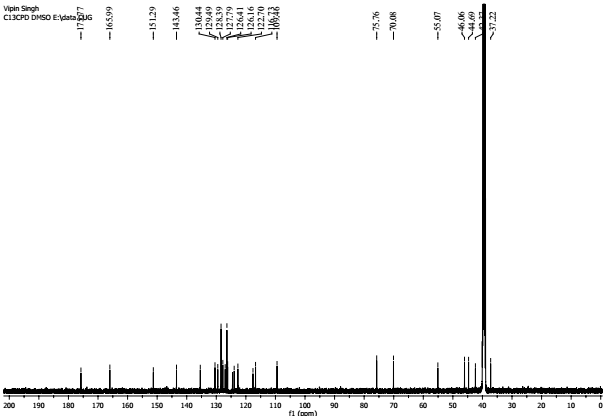


**Figure 3.13** ^1^H NMR (upper) and ^13^C NMR (bottom) of compound **4m**

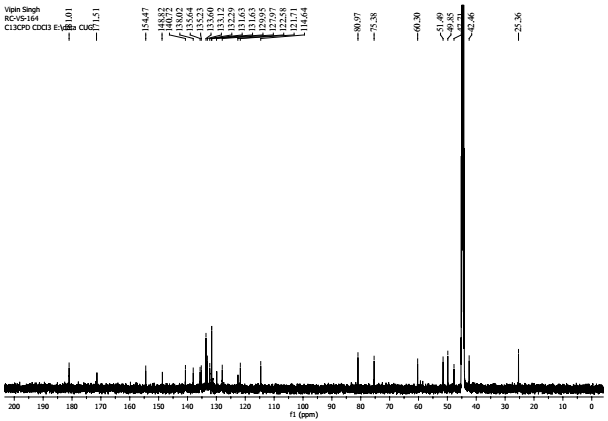


**Figure 3.14** ^1^H NMR (upper) and ^13^C NMR (bottom) of compound **4n**


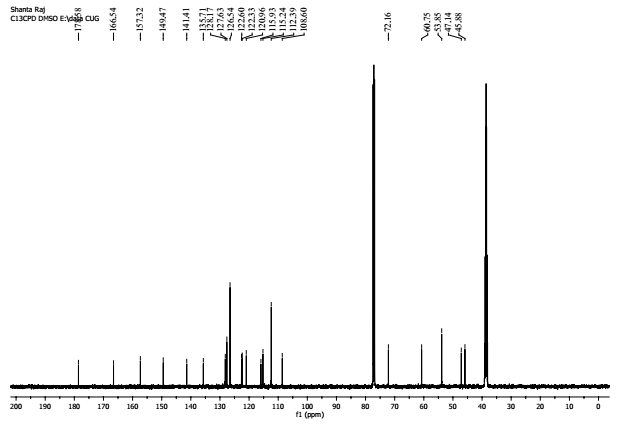


**Figure 3.15** ^1^H NMR (upper) and ^13^C NMR (bottom) of compound **7a**


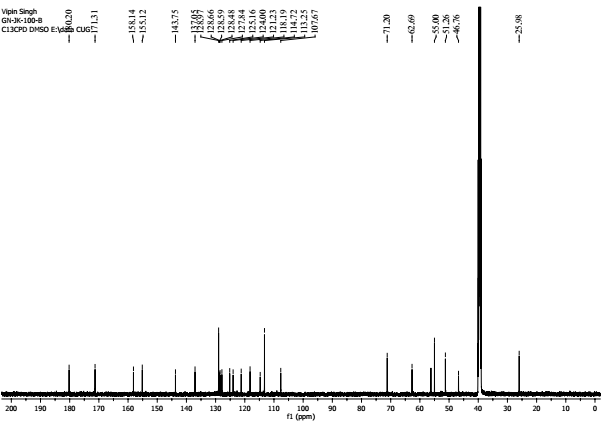


**Figure 3.16** ^1^H NMR (upper) and ^13^C NMR (bottom) of compound **7b**


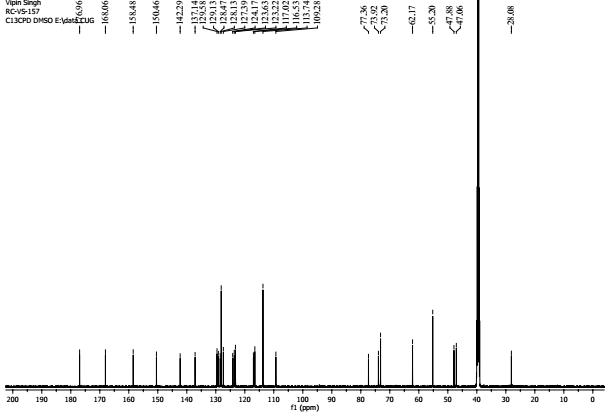


**Figure 3.17** ^1^H NMR (upper) and ^13^C NMR (bottom) of compound **7c**


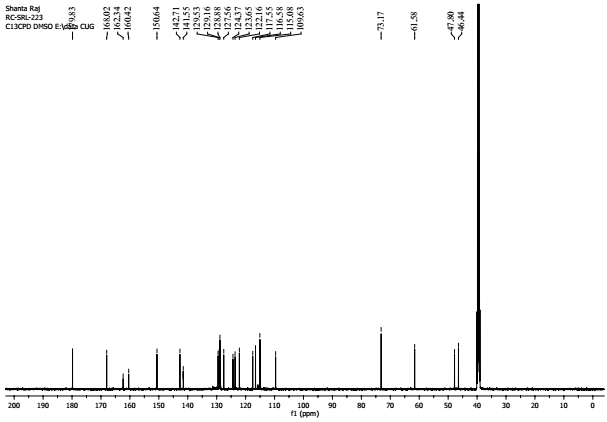
 **Figure 3.18** ^1^H NMR (upper) and ^13^C NMR (bottom) of compound **7d**

**Figure 3.18** ^1^H NMR (upper) and ^13^C NMR (middle) and ^19^F NMR (lower) of compound **7d**


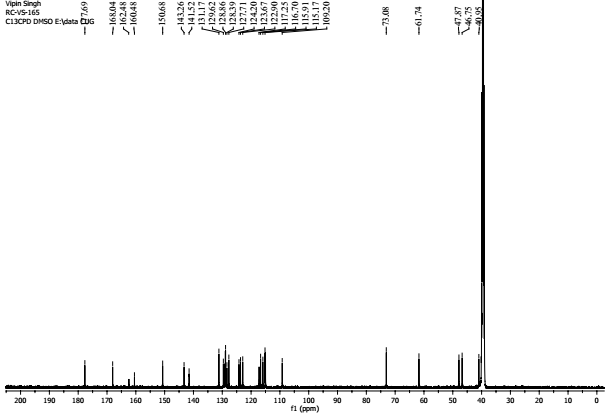

**Figure 3.19** ^1^H NMR (upper) and ^13^C NMR (middle) and ^19^F NMR (lower) of compound **7e**


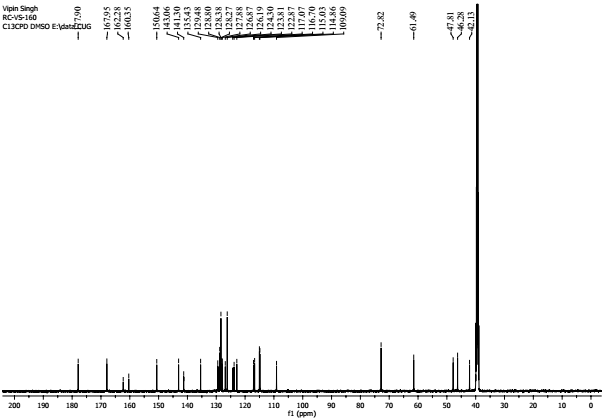

**Figure 3.20** ^1^H NMR (upper) and ^13^C NMR (middle) and ^19^F NMR (lower) of compound **7f**


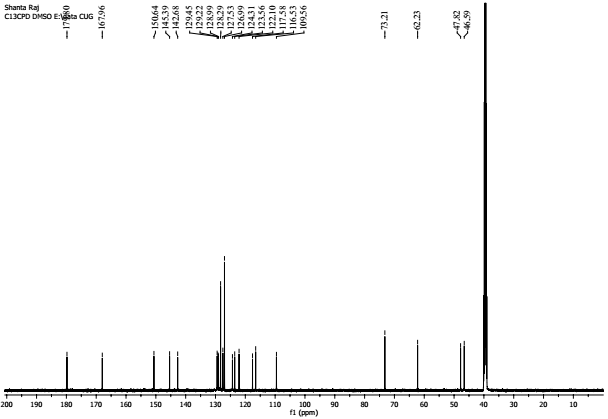


**Figure 4.21** ^1^H NMR (upper) and ^13^C NMR (bottom) of compound **7g**


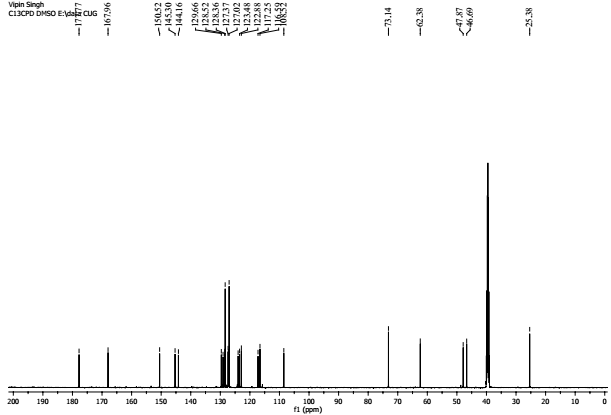
**Figure 3.22** ^1^H NMR (upper) and ^13^C NMR (bottom) of compound 7**h**


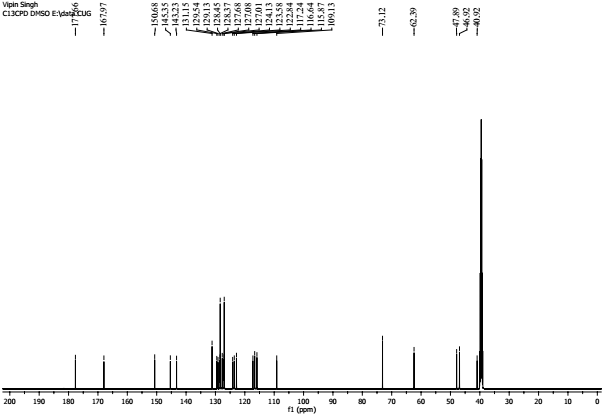


**Figure 3.23** ^1^H NMR (upper) and ^13^C NMR (bottom) of compound **7i**


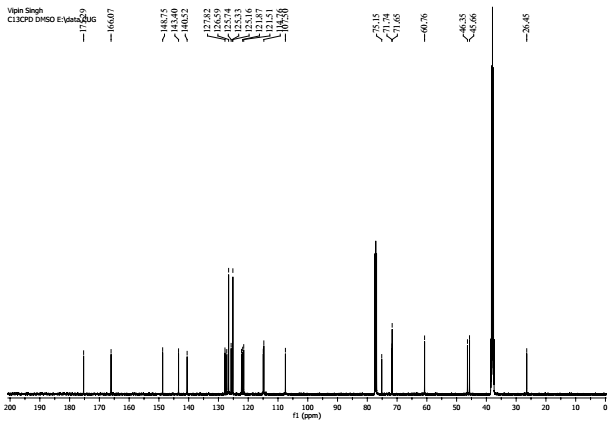


**Figure 3.24** ^1^H NMR (upper) and ^13^C NMR (bottom) of compound **7j**


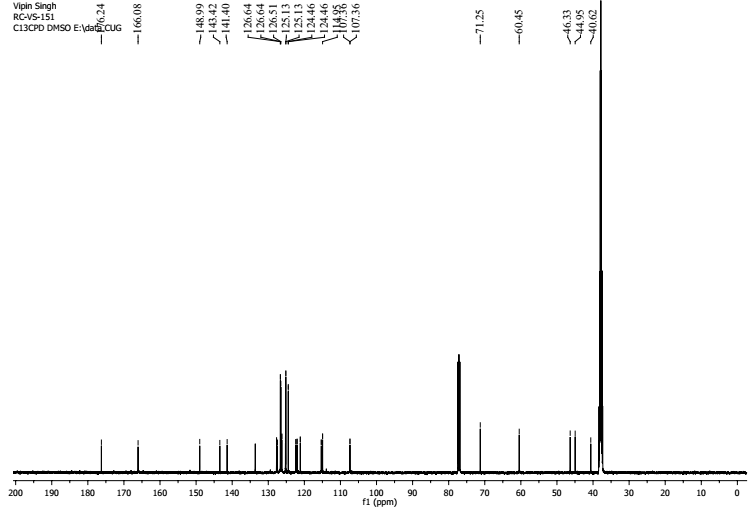
**Figure 3.25** ^1^H NMR (upper) and ^13^C NMR (bottom) of compound **7k**


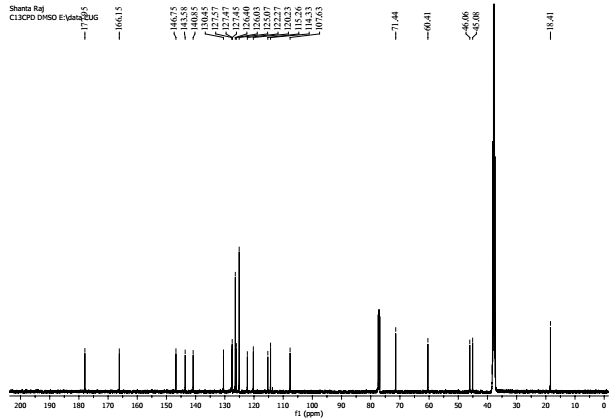


**Figure 3.26** ^1^H NMR (upper) and ^13^C NMR (bottom) of compound **7l**


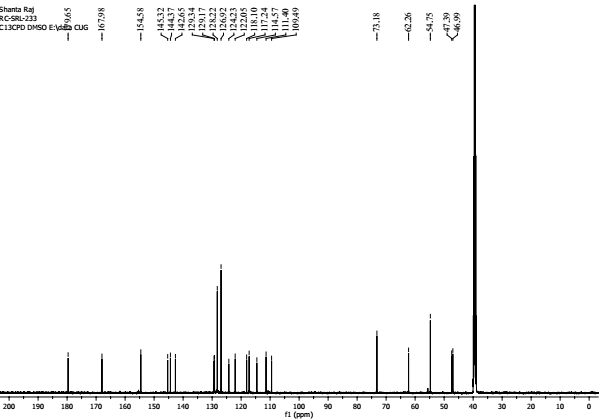


**Figure 3.27** ^1^H NMR (upper) and ^13^C NMR (bottom) of compound **7m**


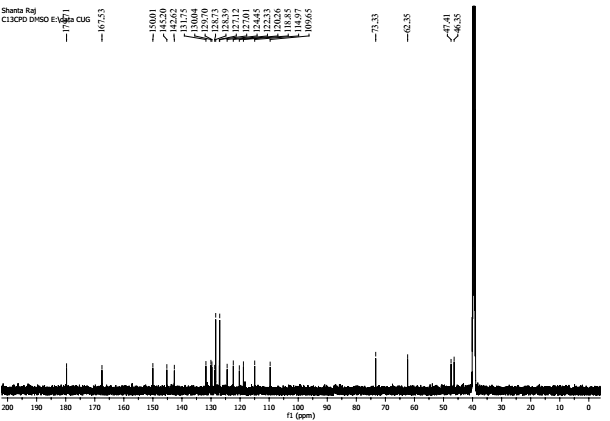


**Figure 3.28** ^1^H NMR (upper) and ^13^C NMR (bottom) of compound **7n**


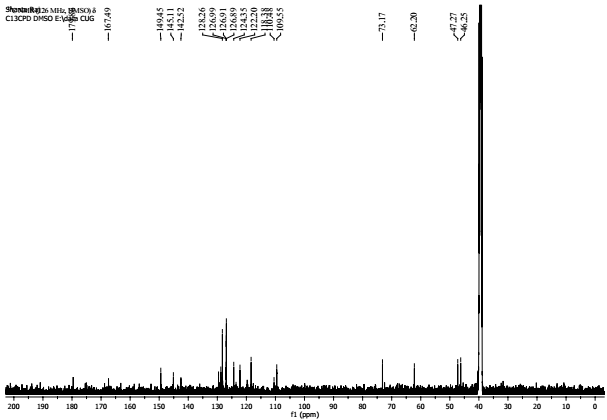


**Figure 3.29** ^1^H NMR (upper) and ^13^C NMR (bottom) of compound **7o**
